# Immunogenicity and protection of a variant nanoparticle vaccine that confers broad neutralization against SARS-CoV-2 variants

**DOI:** 10.1038/s41467-022-35606-6

**Published:** 2023-02-28

**Authors:** James Logue, Robert M. Johnson, Nita Patel, Bin Zhou, Sonia Maciejewski, Bryant Foreman, Haixia Zhou, Alyse D. Portnoff, Jing-Hui Tian, Asma Rehman, Marisa E. McGrath, Robert E. Haupt, Stuart M. Weston, Lauren Baracco, Holly Hammond, Mimi Guebre-Xabier, Carly Dillen, M. Madhangi, Ann M. Greene, Michael J. Massare, Greg M. Glenn, Gale Smith, Matthew B. Frieman

**Affiliations:** 1 The Department of Microbiology and Immunology, The University of Maryland School of Medicine, Baltimore, MD 21201 USA; 2 Center for Pathogen Research, The University of Maryland School of Medicine, Baltimore, MD 21201 USA; 3 Novavax, Inc, 21 Firstfield Road, Gaithersburg, MD 20878 USA; 4 Present Address: Johns Hopkins University, School of Medicine, 720 Rutland Avenue, Ross 1164, Baltimore, MD 21205 USA

**Keywords:** Virology, Vaccines, SARS-CoV-2

## Abstract

SARS-CoV-2 variants have emerged with elevated transmission and a higher risk of infection for vaccinated individuals. We demonstrate that a recombinant prefusion-stabilized spike (rS) protein vaccine based on Beta/B.1.351 (rS-Beta) produces a robust anamnestic response in baboons against SARS-CoV-2 variants when given as a booster one year after immunization with NVX-CoV2373. Additionally, rS-Beta is highly immunogenic in mice and produces neutralizing antibodies against WA1/2020, Beta/B.1.351, and Omicron/BA.1. Mice vaccinated with two doses of Novavax prototype NVX-CoV2373 (rS-WU1) or rS-Beta alone, in combination, or heterologous prime-boost, are protected from challenge. Virus titer is undetectable in lungs in all vaccinated mice, and Th1-skewed cellular responses are observed. We tested sera from a panel of variant spike protein vaccines and find broad neutralization and inhibition of spike:ACE2 binding from the rS-Beta and rS-Delta vaccines against a variety of variants including Omicron. This study demonstrates that rS-Beta vaccine alone or in combination with rS-WU1 induces antibody-and cell-mediated responses that are protective against challenge with SARS-CoV-2 variants and offers broader neutralizing capacity than a rS-WU1 prime/boost regimen alone. Together, these nonhuman primate and murine data suggest a Beta variant booster dose could elicit a broad immune response to fight new and future SARS-CoV-2 variants.

## Abstract (web summary)

Current SARS-CoV-2 variants evade immune responses induced by approved vaccines. In this study, the authors find that a recombinant prefusion-stabilized Beta spike protein vaccine confers broad neutralization capacity in mice and non-human primates as well as protective antibody and immune memory responses.

## Introduction

The emergence of severe acute respiratory syndrome coronavirus 2 (SARS-CoV-2) in late 2019 generated an urgent medical need for an effective vaccine to combat the pandemic. SARS-CoV-2 has infected over 500 million individuals causing over 6 million deaths by mid-2022 (www.who.org). This pandemic has altered economic and social structures throughout the world. Enhanced understanding of coronavirus biology from past outbreaks of SARS-CoV in 2003 and middle east respiratory syndrome coronavirus (MERS-CoV) in 2012 enabled the rapid design and emergency use approval of Coronavirus Disease 2019 (COVID-19) vaccines^1–4^. Global spread of SARS-CoV-2 has been punctuated by the identification of mutant viruses, or variants of concern (VoCs), that have gained function as they spread. As coronaviruses replicate, they have a 1 in 100,000 chance of mutating a base in their genomes due to the high fidelity of the RNA-dependent RNA polymerase encoded in the virus^5,6^. When selective pressure is placed on the virus, mutations emerge that facilitate improved receptor binding, replication to higher titers resulting in enhanced transmission, or immune evasion, and the variants that emerge from these bottlenecks have spread with the potential to evade current countermeasures^7^. Two SARS-CoV-2 VoCs emerged in 2021, one first identified in the United Kingdom called Alpha (or given the PANGO name of B.1.1.7) and the other in the Republic of South Africa called Beta (or given the PANGO name of B.1.351)^8,9^. Alpha was transmitted more readily between individuals than the ancestral strain, causing increased hospitalizations and prevalence around the world^1^. Beta spread to many countries; it rapidly became among the most prevalent circulating strains^10^. Alpha has been shown to be neutralized by SARS-CoV-2 convalescent sera comparably to its ancestral virus; however, Beta gained the ability to evade several authorized monoclonal antibodies and shows reduced neutralization by convalescent sera^10–12^. Since that time, additional variants of concern have emerged with mutations that could lead to partial immune evasion, namely the Delta (B.1.617.2) and Omicron (B.1.1.529, BA.1, BA.5) variants (ww.who.org).

A Phase 3 trial in the United Kingdom using Novavax’s prototype SARS-CoV-2 vaccine, NVX-CoV2373 (based on the Wuhan-1 strain, rS-WU1), demonstrated it to be highly effective (86.3%) at preventing infection by the highly transmissible Alpha strain that was circulating during the trial^13,14^. A Phase 2b trial of NVX-CoV2373 in South Africa showed this vaccine’s reduced efficacy against the Beta strain (60.1% in HIV-negative subjects); this decreased efficacy against the Beta strain has also been observed for other SARS-CoV-2 vaccines^15^. In response to these breakthrough transmission events, we developed a full-length, stabilized recombinant spike protein (rS) antigen with mutations in the Beta variant (rS-Beta). We evaluated cellular and humoral immunogenicity and protective efficacy of rS-Beta alone or in combination with our prototype vaccine based on Wuhan-Hu-1 (rS-WU1). B and T cells were highly activated by rS-Beta vaccination leading to the production of neutralizing antibodies and expansion of multifunctional T cells. Immunization with rS-Beta induced high levels of neutralizing antibodies against multiple variant strains of SARS-CoV-2 as well as the parent USA-WA1/2020 strain. Mice immunized with either rS-Beta or rS-WU1, then challenged with the Alpha or Beta strain, showed a 5-log reduction in Beta virus titers and 2-log reduction of Alpha (which replicates to lower levels than Beta in mice), both to lower than the limit of detection. All immunized mice were protected against challenge-induced weight loss when infected with the Beta strain. In olive baboons subjected to a primary immunization series with rS-WU1, boosting with rS-Beta approximately 1 year later led to a robust anamnestic response, with the rapid induction of anti-spike IgG and functional antibody responses and spike-specific T-cell responses. Finally, immunization with rS vaccines based on several VoCs resulted in robust cross-neutralizing antibody responses in mice, acting as a proof-of-concept that variant spike-based SARS-CoV-2 vaccines can provide broad protection against emerging variants.

In this work, we demonstrate the development and evaluation of a Beta spike-directed variant vaccine that induced highly potent neutralizing antibodies, activated both B and T cells, protected mice from clinical disease, and prevented detectable SARS-CoV-2 replication in the lungs. Sera from Beta spike-vaccinated BALB/c laboratory mice demonstrated broad neutralization against variants, even those that emerged after it, as compared to the prototype vaccine. Similar results in nonhuman primates also suggest the feasibility of utilizing a Beta variant vaccine as a booster to broaden the neutralizing antibody response to new SARS-CoV-2 variants yet to emerge.

## Results

### Production of spike glycoprotein vaccine rS-Beta

**Fig. 1:**
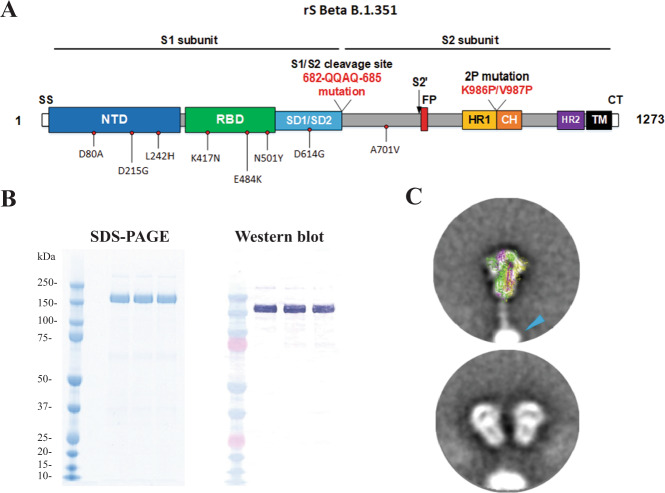
Characterization of SARS-CoV-2 recombinant spike protein construct based on Beta variant. **A** Linear diagram of the full-length SARS-CoV-2 spike (S) protein based on the protein sequence of the Beta variant. Structural elements include the cleavable signal sequence (SS, white), N-terminal domain (NTD, blue), receptor binding domain (RBD, green), subdomains 1 and 2 (SD1 and SD2, cyan), S2 cleavage site (S2ʹ), fusion peptide (FP, red), heptad repeat 1 (HR1, yellow), central helix (CH, orange), heptad repeat 2 (HR2, purple), transmembrane domain (TM, black), and cytoplasmic tail (CT, white). Amino acid changes from the prototype rS protein sequence (rS-WU1) are shown in black text underneath the linear diagram. The native furin cleavage site was mutated (RRAR to QQAQ) to resist proteolytic cleavage and two proline mutations were also introduced to increase stability; these mutations are noted in red text underneath the linear diagram. **B** Reduced SDS-PAGE gel with Coomassie blue staining of purified full-length rS-Beta construct (left panel) showing the main protein product at the expected molecular weight of ~170 kD. Western blot using a spike-specific primary antibody confirming the identity of the main protein product (center panel). Scanning densitometry results are shown in the right panel. **C** Negative stain transmission electron microscopy and 2D class averaging of rS-Beta. 2D images of rS-Beta showed well-defined lightbulb-shaped intact prefusion spike trimeric particles. Trimers exhibited as a nanoparticle with PS-80 micelles as indicated with a cyan arrow in the top panel. Class average images showed a good fit of the rS-Beta trimer with a cryo-EM solved the structure of the prefusion SARS-CoV-2 trimeric spike protein ectodomain (PDB ID 6VXX) overlaid on the 2D image (top panel). The bottom panel shows two rS-Beta trimers anchored into a PS-80 micelle.

The antigen component of our prototype vaccine, NVX-CoV2373, was based on the spike glycoprotein from the SARS-CoV-2/Wuhan-Hu-1 sequence (now the B.2 lineage; MN908947.3) with mutations: 682-RRAR-685 in the furin cleavage site was mutated to QQAQ to resist proteolytic cleavage (“3Q” mutation), plus K986P and V987P were introduced to increase stability (“2P” mutations). The spike ORF was cloned into recombinant baculovirus and expressed in Sf9 insect cells as previously described^16^. NVX-CoV2373 is currently authorized or approved for adult use in over 40 countries, including the USA, Mexico, and UK. In addition, this vaccine technology does not utilize human fetal or embryonic cells during vaccine testing or manufacturing, nor are human fetal or embryonic cells or tissue contained in the vaccine. Using an identical expression and purification system with the same 3Q-2P mutations, we have also developed a spike glycoprotein vaccine based on the spike ORF from the SARS-CoV-2 Beta B.1.351 lineage that emerged in South Africa, also containing the 3Q-2P mutations (Fig. 1A). This recombinant spike protein (rS-Beta) was expressed, purified, and formulated with Matrix-M^TM^ adjuvant.

### Biophysical properties, structure, and function of the rS-B.1.351 antigen

Purified SARS-CoV-2 recombinant spike (rS) protein rS-Beta, when reduced and subjected to SDS-PAGE, migrated with the expected molecular weight of ~170 kDa (Fig. 1B). The thermal stability of rS-Beta was compared to that of rS-WU1 by differential scanning calorimetry (DSC); the main peak of the rS-Beta protein showed a 4.7 °C increase in thermal transition temperature (*T*_max_) and 1.2-fold higher enthalpy of transition (ΔHCal) compared to the prototype rS-WU1 protein, indicating increased stability of the Beta variant construct (Table 1). Negative stain transmission electron microscopy (TEM) imaging combined with two rounds of two-dimensional (2D) class averaging of 16,049 particles were used to confirm the ultrastructure of rS-Beta. High-magnification (×92,000 and ×150,000) TEM images revealed a lightbulb-shaped particle appearance which was consistent with the prefusion form of the SARS-CoV-2 spike trimer (PDB ID 6VXX; Fig. 1C). This is consistent with what we have previously observed for the prototype rS-WU1 spike protein^16^.

**Table 1:** Thermostability and hACE2 binding of SARS-CoV-2 recombinant spike proteins

SARS-CoV-2 rS proteins	Differential scanning calorimetry (DSC)		hACE2 binding		
	*T*_max_ (°C)	ΔHcal (kJ mol^−1^)	hACE2 binding kinetics by bio-layer interferometry		hACE2 ELISA (EC_50_, ng/mL)
			K_a_ (1/Ms)	K_dis_ (1/s)	
rS-Beta	67.88	1883.0	3.94 × 10^4^	1.46 × 10^−7^	8.0
rS-WU1	63.21	1578.9	1.08 × 10^4^	1.56 × 10^−7^	9.4

T_*max*_ melting temperature, *K*_*a*_ binding constant, *K*_*dis*_ dissociation constant, *EC*_*50*_ half-maximal binding.

To confirm the functional properties of the variant spike protein construct rS-Beta, the binding of this rS to the hACE2 receptor was determined using bio-layer interferometry (BLI) as previously described^16^. rS-Beta was found to bind tightly and stably to hACE2, with an association constant (Ka) of 3.94 × 10^4^, representing a 3.6-fold greater association to hACE2 compared to the prototype protein rS-WU1 (Ka = 1.08 × 10^4^). The dissociation constants of these two proteins were essentially identical (1.46 × 10^−7^ and 1.56 × 10^−7^ for rS-Beta and rS-WU1, respectively). We additionally assessed rS-Beta binding to hACE2 with an ELISA as previously described^16^. In this assay, rS-Beta attained 50% saturation of hACE2 at a slightly lower concentration (EC_50_ = 8.0 ng/mL) than the prototype construct rS-WU1 (EC_50_ = 9.4 ng/mL), confirming that the binding affinity of rS-Beta variant spike for hACE2 was comparable to that of rS-WU1 (Table 1).

### Anamnestic response induced by boosting with rS-Beta 1 year after primary immunization with rS-WU1 in olive baboons

**Fig. 2:**
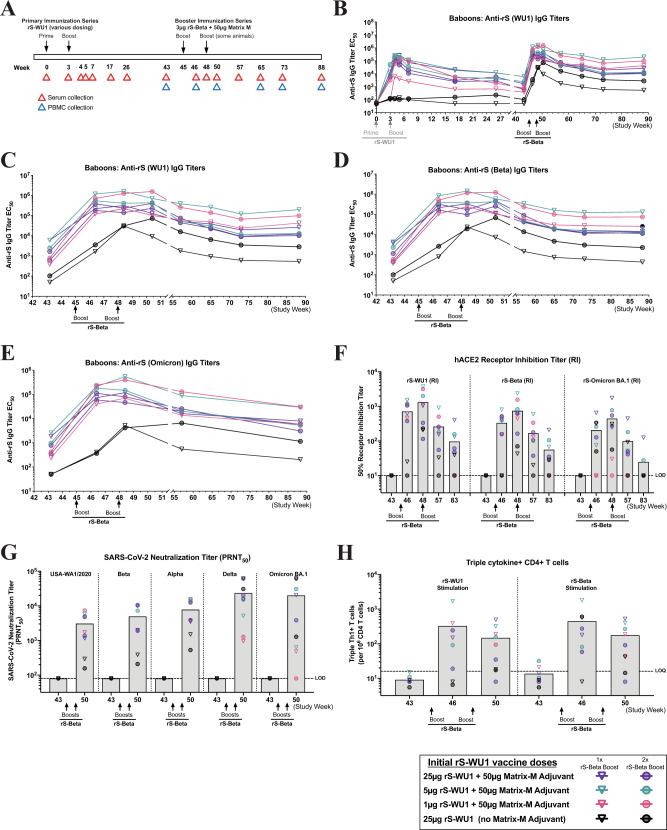
Immunogenicity of one or two booster rS-Beta doses approximately 1 year after immunization with rS-WU1 in olive baboons. **A** A small cohort of baboons (*N* = 2–3/group) were originally immunized with 1 µg, 5 µg, or 25 µg rS-WU1 with 50 µg Matrix-M adjuvant or unadjuvanted 25 µg rS-WU1 on day 0 and 21 (week 0 and 3, respectively). Approximately 1 year later, all animals were boosted with one or two doses of 3 µg rS-Beta with 50 µg Matrix-M adjuvant on day 318 and 339 (week 45 and 48, respectively). **B** Anti-S (WU1) IgG titers were measured throughout the course of the study. Individual animals’ titers are shown over time, different colored symbols and lines represent different dose groups for the initial rS-WU1 immunization series. Sera collected pre-rS-Beta boost (study week 43) as well as 7, 21, 35, 81, 137, 193, and 300 days after the first rS-Beta boost were analyzed to determine **C** anti-rS-WU1 (same data displayed in panel B), **D** anti-rS-Beta, and **E** anti-rS-Omicron BA.1 IgG titers by ELISA (*n* = 3). **F** Antibody titers capable of disrupting the interaction between rS-WU1, rS-Beta, or rS-Omicron and the hACE2 receptor by ELISA (*n* = 3) (gray bars represent means), and **G** antibody titers capable of neutralizing SARS-CoV-2 variants USA-WA1, Beta, Alpha, Delta, and Omicron with a PRNT assay (*n* = 3) (gray bars represent geometric means). **H** The presence of multifunctional CD4 + T cells positive for three Th1 cytokines (IFN-γ, IL-2, and TNF-α) was evaluated with intracellular cytokine staining after stimulation with rS-WU1 or rS-Beta (*n* = 2, gray bars represent means).

A small cohort of olive baboons (*N* = 9 total) were subjected to a primary immunization series with rS-WU1 (either 1 µg, 5 µg, or 25 µg rS with 50 µg Matrix-M adjuvant or unadjuvanted 25 µg rS), as described previously^16^. Approximately 1 year later, all animals were boosted with one or two doses of 3 µg rS-Beta with 50 µg Matrix-M adjuvant to examine the resulting immune responses (Fig. 2A and Supplementary Table S1). Seven days after the first rS-Beta boost, animals that had originally received adjuvanted rS-WU1 exhibited a robust anamnestic response as exhibited by levels of anti-S (WU1) IgG titers higher than that originally observed at peak immune response during the primary immunization series (Fig. 2B). This response did not seem to be further bolstered by a second booster dose of rS-Beta, though the small sample sizes utilized in this study prohibit a meaningful quantitative analysis. Animals that received unadjuvanted rS-WU1 during the primary immunization series exhibited a weaker response to boosting with rS-Beta, though still exhibited elevated anti-S (WU1) IgG response. The rS-Beta boost elicited comparable antibody titers against rS-WU1 and rS-Beta, with slightly lower titers against rS-Omicron (BA.1), and animals that originally received unadjuvanted rS-WU1 exhibited a weaker response (Fig. 2E).

Serum antibody titers capable of disrupting the interaction between rS-WU1, rS-Beta, or rS-Omicron BA.1 and hACE2 were also evaluated before an rS-Beta boost (study week 43), and at 7, 21, 89, and 300 days after the initial rS-Beta boost (study weeks 46, 48, 57, and 83, dosing strategy in Supplementary Table S1). Similar to what was observed for anti-S IgG titers, animals that had received an adjuvanted vaccine during the primary immunization series exhibited a strong hACE2-inhibiting antibody response seven days after the rS-Beta boost, despite having undetectable titers before the boost. Titers were slightly higher for rS-WU1:hACE2 blocking antibodies compared to levels of rS-Beta:hACE2 blocking antibodies and rS-Omicron-BA.1:hACE2 blocking antibodies, though the small sample size prohibits a meaningful quantitative analysis. Animals that had received an unadjuvanted vaccine during the primary immunization series exhibited lower hACE2 blocking titers after the rS-Beta boost (Fig. 2F).

Neutralizing antibody titers were analyzed by Plaque Reduction Neutralization Titer (PRNT) assay by testing sera for the ability to neutralize USA-WA1/2020, Beta, Alpha, Delta, and Omicron BA.1. Sera collected just before the rS-Beta boost (study week 43) had undetectable neutralizing antibody levels against all of these viruses. By 35 days post vaccination (study week 50), high antibody titers that neutralized all five strains were detected, and this antibody response stayed high through 35 days post vaccination. Animals immunized with unadjuvanted rS-WU1 in the primary series displayed significantly lower antibody levels with a much broader range of neutralization titers (Supplementary Fig. 2G). Together, these data demonstrate a robust, durable antibody response even 1 year after the primary vaccination series.

In addition to humoral responses, functional T-cell responses were assessed by collection of PBMCs and testing by ELISpot and intracellular cytokine staining (ICS) to examine cytokine secretion upon stimulation with rS-WU1 or rS-Beta. We observed multifunctional antigen-specific CD4^+^ T cells producing three Th1 cytokines upon rS-WU1 or rS-Beta stimulation were expanded 7 days after the first rS-Beta booster dose in baboons (study week 46). These responses were maintained at 35 days after the first booster dose (study week 50, Fig. 2H and Supplementary Fig. S1).

### rS-Beta vaccine immunogenicity in BALB/c laboratory mice

**Fig. 3:**
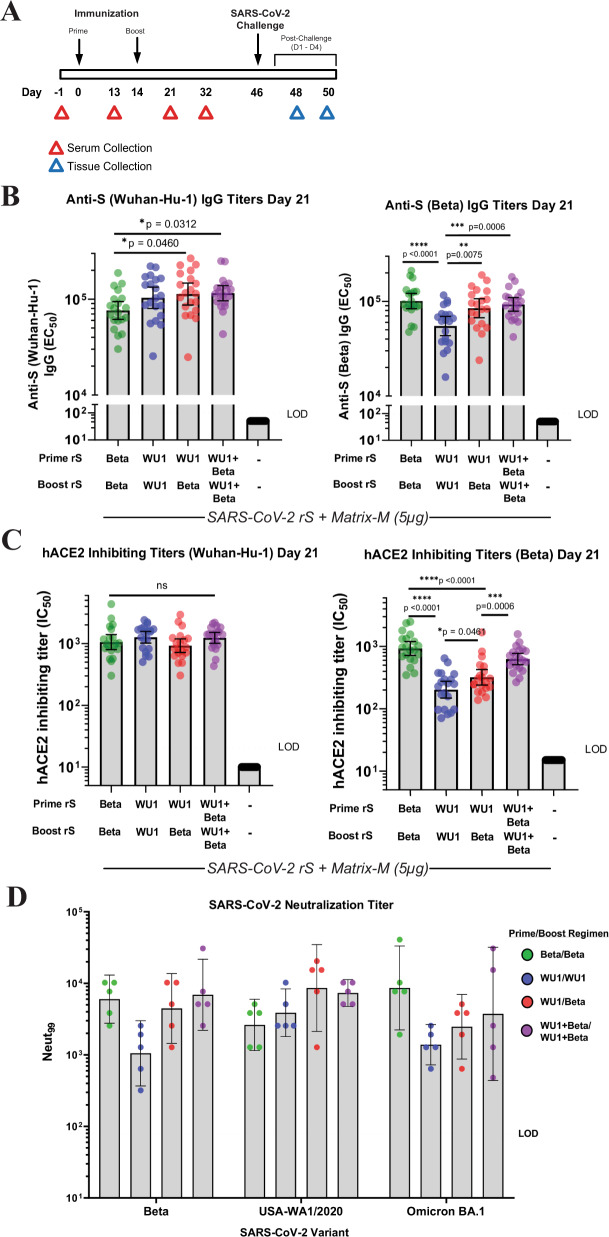
Antibody-mediated immunity induced upon immunization with SARS-CoV-2 rS based on Wuhan-Hu-1 or Beta variant in BALB/c laboratory mice. **A** Groups of mice (*N* = 20/group) were immunized in a prime/boost regimen on days 0 and 14 with combinations of SARS-CoV-2 rS based on Beta or Wuhan-Hu-1. Mice were either primed and boosted with rS-Beta, with rS-WU1, primed with rS-WU1 and boosted with rS-Beta, or primed and boosted with bivalent rS-WU1 + rS-Beta. Antigen doses were 1 µg rS for each monovalent immunization, or 1 µg rS for each bivalent immunization (2 µg rS total). All antigen doses were administered with 5 µg Matrix-M adjuvant. A control group received a formulation buffer (Placebo). Sera and tissues were collected at the timepoints listed in the diagram. **B** Anti-SARS-CoV-2 S IgG serum titers were measured in sera collected on day 21 using an ELISA to measure antibody titers against the Wuhan-Hu-1 spike protein (left panel) or Beta spike protein (right panel) (*n* = 20). Bars indicate the geometric mean titer (GMT) and error bars represent 95% confidence interval (CI) for each group. Individual animal titers are indicated with colored symbols. **C** ELISA was also used to determine the functional antibody titers in sera collected on day 21 capable of disrupting binding between the SARS-CoV-2 receptor hACE2 and Wuhan-Hu-1 spike protein (left panel) or Beta spike protein (right panel) (*n* = 20). Bars indicate the geometric mean titer (GMT) and error bars represent 95% confidence interval (CI) for each group. Individual animal titers are indicated with colored symbols. **D** SARS-CoV-2 neutralization antibody titers in sera collected on day 32 from *n* = 5 animals/group were determined using ny microneutralization. Sera were evaluated for neutralization of SARS-CoV-2 USA-WA1, Beta, or Omicron (*n* = 5 samples per group, run in duplicate). Bars indicate the geometric mean titer (GMT) and error bars represent 95% confidence interval (CI) for each group. Individual animal titers are indicated with symbols. Statistical significance was calculated by performing one-way ANOVA with Tukey’s post hoc test on log_10_-transformed data; significant differences among groups are indicated with asterisks and *P* values in panels B and C.

We assessed the antibody- and cell-mediated immunogenicity of rS-Beta and rS-WU1 (Wuhan-Hu-1 spike) formulated with Matrix-M adjuvant. Study design for these experiments was consistent with previously published testing of our rS-WU1 vaccine in BALB/c laboratory mice^16^. To assess antibody-mediated immunogenicity, groups of mice (*N* = 20) were immunized with either rS-WU1 or rS-Beta as both prime and boost (homologous), with rS-WU1 as the prime and rS-Beta as the boost (heterologous), or with both vaccines combined in a bivalent formulation for the prime and boost vaccination. A placebo group received vaccine formulation buffer as a negative control. In monovalent immunization groups, 1 µg of rS formulated with 5 µg of Matrix-M adjuvant was intramuscularly injected at days 0 and 14. For bivalent immunization, 1 µg of each rS construct was administered at each immunization, for a total of 2 µg rS, with 5 µg of Matrix-M adjuvant. The study design is shown in Fig. 3A. Mice immunized with either of the four vaccine regimens displayed elevated antibody titers against both the WU1 spike and Beta spike by ELISA at day 21 post vaccination. Bivalent vaccination or heterologous vaccination produced significantly higher anti-S (WU1) IgG titers than monovalent vaccination with rS-Beta, although group differences in IgG titers remained within twofold or less (Fig. 3B). Regarding IgG titers against Beta spike, immunization with monovalent rS-Beta or bivalent rS resulted in anti-Beta Spike IgG titers that were the highest among regimens tested, with no significant difference between these regimens. Immunization with rS-WU1 alone resulted in significantly lower titers against Beta spike compared to all other immunization regimens (Fig. 3B). Animals in the placebo group exhibited undetectable anti-WU1 spike and anti-Beta spike IgG titers as expected.

The ability of serum from mice to inhibit spike binding to hACE2 was also assessed (Fig. 3C). All immunization regimens resulted in the production of antibodies that blocked hACE2 binding to WU1 spike with no significant difference between any groups at day 21. Yet immunization with rS-WU1 alone resulted in significantly lower serum titers capable of disrupting binding between Beta spike and hACE2; titers in the rS-Beta alone immunization group were 4.6-fold higher than titers in the rS-WU1 alone immunization group (*P* < 0.0001) and titers in the group that received bivalent rS were 3.1-fold higher than titers in the rS-WU1 alone group (*P* < 0.0001). Monovalent rS-Beta immunization or bivalent rS immunization also resulted in significantly higher titers than heterologous immunization (*P* < 0.0001 and *P* = 0.0006, respectively).

We next assessed neutralizing antibody titers among the different vaccination regimens (Fig. 3D). Sera collected from vaccinated animals at day 32 post vaccination were assessed using SARS-CoV-2 USA-WA1/2020, Beta, and Omicron BA.1 strains in a microneutralization assay (Neut_99_). When rS-WU1 was used for the prime and boost, we see a similar pattern that has been reported with high neutralizing antibody titer against WA1 and reduced neutralization for Beta and especially BA.1. Compared to the monovalent immunization with rS-WU1, immunization with monovalent rS-Beta produced elevated neutralizing antibody titers to the Beta and the Omicron BA.1 variants. When rS-WU1 spike is used for the prime and rS-Beta is used for the boost, we find that there is a high level of neutralization against WA1 and increased neutralization for BA.1. When rS-WU1 and rS-Beta are combined for the prime and boost vaccinations, we see heightened neutralization of WA1 and BA.1, demonstrating broad neutralizing capacity when rS-Beta is included in the vaccination (Fig. 3D).

### rS-Beta protection against SARS-CoV-2 in BALB/c laboratory mice

**Fig. 4:**
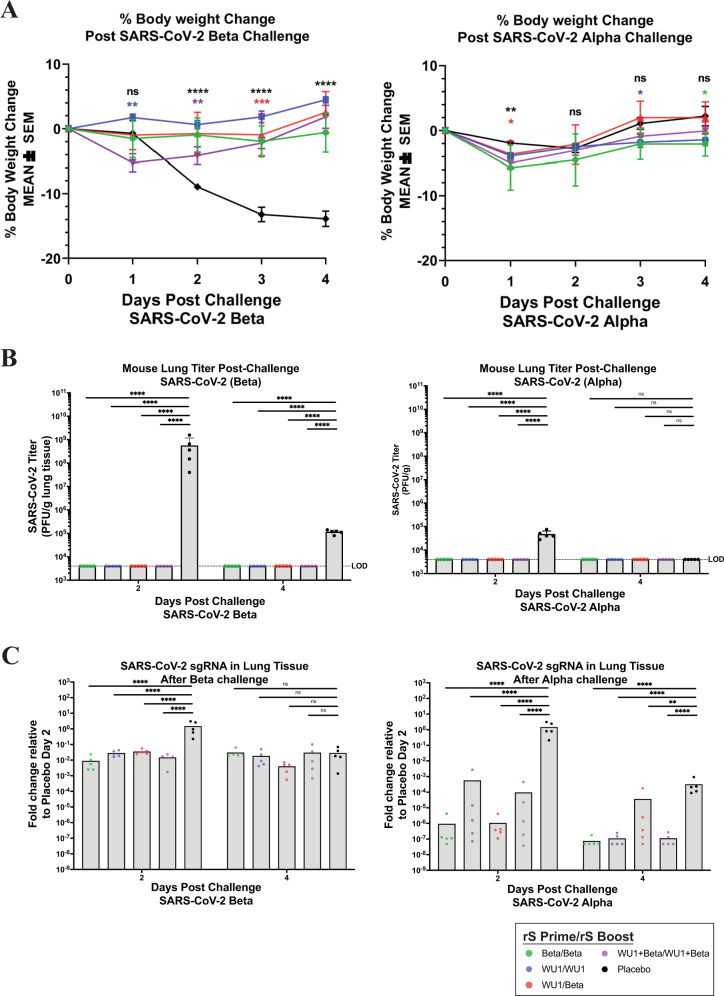
Protective efficacy of immunization with SARS-CoV-2 rS based on Wuhan-Hu-1 or Beta against challenge with live SARS-CoV-2 Beta or Alpha virus in BALB/c laboratory mice. Mice were immunized in a prime/boost regimen on days 0 and 14 with combinations of SARS-CoV-2 rS based on Beta or Wuhan-Hu-1. Mice were either prime/boosted with rS-Beta, primed/boosted with rS-WU1, primed with rS-WU1 and boosted with rS-Beta, or prime/boosted with bivalent rS-WU1 + rS-Beta. Antigen doses were 1 µg rS for each monovalent immunization or 1 µg rS for each construct upon bivalent immunization (2 µg rS total). All immunizations were administered with 5 µg Matrix-M adjuvant. A control group received formulation buffer (Placebo, *N* = 5). Immunized mice (*N* = 10/group) were challenged with SARS-CoV-2 Beta (left panels) or Alpha (right panels). For 4 days after challenge, mice were weighed daily and their percentage weight loss was calculated relative to their initial body weight. **A** Mean percentage body weight loss is shown with symbols and error bars represent standard error of the mean. Student’s *t* test was used to calculate significance of differences between each immunization group and the placebo group groups. ns nonsignificant; *P < 0.05; **P < 0.001, ***P < 0.005; ****P < 0.0001. Colors indicate the respective immunization group being compared; differences between two or more immunization groups and placebo group are shown in black. **B** Half of the mice were sacrificed at 2 days post-challenge and lung tissue was subjected to a plaque formation assay to determine lung viral titers, the remaining mice were sacrificed at 4 days post-challenge. Mean and standard deviation of lung titer are graphed. **C** Levels of SARS-CoV-2 subgenomic RNA were also determined in lung tissue and expressed as fold change in RNA relative to the mean in the respective Placebo group on day 2 post-challenge (*n* = 5 mice per group run in duplicate). Horizontal bars represent group mean fold change from *N* = 5 mice at each timepoint and error bars represent standard deviation. For **B**, **C**, mixed-effects analysis was used to compare differences in viral loads from lung homogenates between vaccinated groups and the placebo control group; **P ≤ 0.01, ***P ≤ 0.001, ****P ≤ 0.0001.

Mice vaccinated as described in Fig. 3A were challenged with either SARS-CoV-2 Alpha or Beta to evaluate the protective efficacy elicited by immunization. While the SARS-CoV-2/Wuhan-Hu-1 strain does not replicate in wild-type mice, the Alpha and Beta variants have a 501Y mutation in the spike open reading frame (ORF), allowing the spike protein to bind to mouse ACE2 and enter cells. The Beta variant replicates very efficiently in mice while the Alpha variant replicates several logs lower in lungs at the same timepoints. The Alpha variant is used as a “wild-type” comparator in these studies. At day 46 post vaccination, mice were intranasally inoculated with either 7 × 10^4^ PFU of Alpha (*N* = 10 mice per group) or 1 × 10^5^ PFU of Beta (*N* = 10 mice per group). Mice were weighed daily throughout the post-challenge period, and at 2- and 4 days post infection (Study days 48 and 50), five mice per group were euthanized by isoflurane inhalation. Lungs from each mouse were then assessed for viral load by plaque formation assay and viral RNA by RT-qPCR. Placebo BALB/c laboratory mice infected with the Alpha strain did not lose weight and there was also no observed weight loss in any vaccinated groups following infection. For Beta-infected mice, 20% weight loss was observed in the placebo vaccination group by day four post infection (Fig. 4A). All mice vaccinated with either regimen were protected from weight loss after infection with Beta, demonstrating a clinical correlate of protection in this model.

At day 2 post infection, Alpha-infected mice in the placebo group exhibited 4 × 10^4^ pfu/g lung, which dropped to undetectable levels by day 4 post infection in the placebo vaccinated group. Peak titer of virus in lungs of Alpha-infected mice is several logs lower than Beta-infected mice. Upon immunization with any rS-WU1 or rS-Beta regimen, there was no detectable live virus at day 2 or day 4 post infection, demonstrating a greater than 2-log reduction in viral load and protection from infection following vaccination (Fig. 4B). At day 2 post infection, Beta-infected mice in the sham vaccinated group exhibited 2.96 × 10^8^ pfu/g lung, which dropped to 2 × 10^5^ pfu/g lung by day 4 post infection. Upon immunization with any rS regimen, there was no detectable live virus at day 2 or day 4 post infection in the Beta-infected mice. This demonstrates a dramatic reduction in virus titer, with >5 log reduction in viral load by day two post infection from the sham vaccinated mice (Fig. 4B). Lung RNA was also assayed for subgenomic SARS-CoV-2 mRNA (sgRNA) production after challenge. Relative to levels in the respective placebo groups, we found >99% reduction in lung sgRNA levels in immunized mice at day 2 after infection with either Alpha or Beta (Fig. 4C).

These results confirm that rS-WU1 and rS-Beta formulated with Matrix-M adjuvant and administered as monovalent, bivalent, or heterologous regimens confer protection against both strains of SARS-CoV-2, Alpha and Beta, in mice. The less efficient replication of the Alpha variant in mice reduces the power of in vivo comparison. Together with the reduction in weight loss, high neutralizing antibody titers, and elimination of viral replication in the lungs of mice, we demonstrate a highly protective vaccine response by the variant spike targeted vaccine.

### Cell-mediated immunogenicity of rS-Beta in BALB/c laboratory mice

**Fig. 5:**
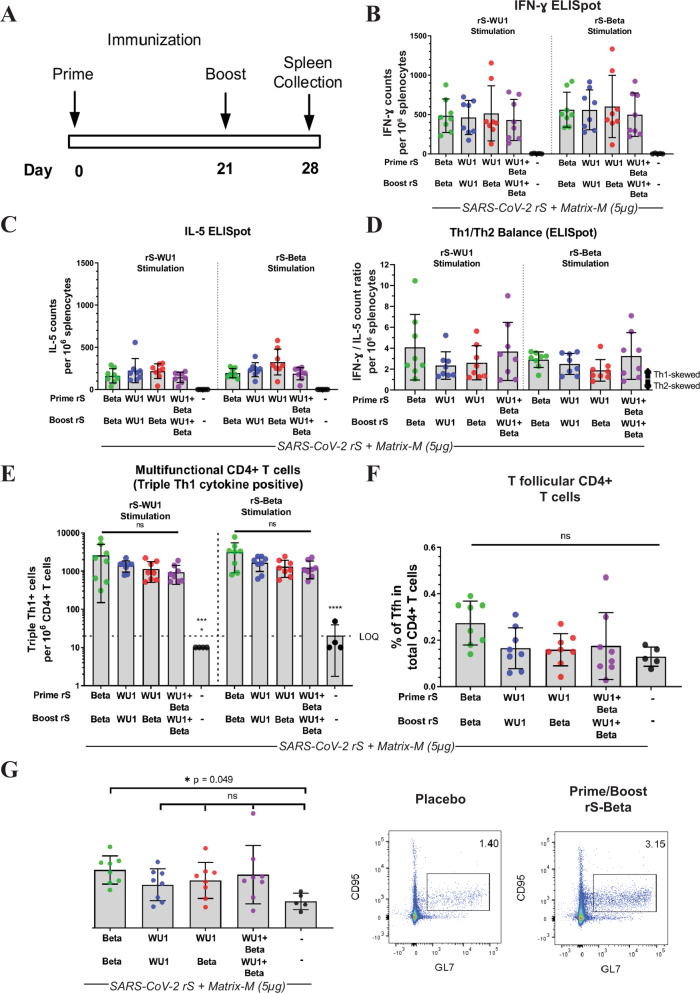
Cell-mediated immunity upon immunization with rS-WU1 or rS-Beta regimens in BALB/c laboratory mice. **A** Groups of mice (*N* = 8/group) were immunized in a prime/boost regimen on days 0 and 21 with various combinations of SARS-CoV-2 rS based on Beta or Wuhan-Hu-1. Mice were either primed and boosted with rS-Beta, primed and boosted with rS-WU1, primed with rS-WU1 and boosted with rS-Beta, or primed and boosted with bivalent rS-WU1 + rS-Beta. Antigen doses were 1 µg rS for each monovalent immunization, or 1 µg rS for each construct upon bivalent immunization (2 µg rS total). All immunizations were administered with 5 µg Matrix-M adjuvant. A control group received formulation buffer (Placebo, *n* = 5). **B**, **C** Spleens were harvested on day 28 for cell collection. Splenocytes were stimulated with rS-WU1 or rS-Beta, then subjected to ELISpot assay in triplicate to determine IFN-γ-positive cells as a representative Th1 cytokine (**B**) and IL-5-positive cells as a representative Th2 cytokine (*n* = 8) (**C**). Data from panels B and C were used to calculate the Th1/Th2 balance of responses to immunization (**D**). **E** The numbers of multifunctional CD4 + T cells that stained positively for three Th1 cytokines (IFN-γ, IL-2, and TNF-α) using intracellular cytokine staining were quantified and expressed as the number of triple cytokine-positive cells per 10^6^ CD44^hi^CD62^Low^ effector memory CD4 + T cells (*n* = 8). **F** T-follicular helper cells were quantified by determining the percentage of PD-1 + CXCR5 + cells among all CD4 + T cells (*n* = 8). **G** Germinal center formation was evaluated by determining the percentage of GL7 + CD95 + cells among CD19 + B cells using flow cytometry (*n* = 8). Gray bars represent means and error bars represent standard deviation. Individual animal data are shown with colored symbols. An example of the gating strategy is shown in the right panel. Mean and standard deviation are graphed in B-G. Differences among experimental groups were evaluated by one-way ANOVA with Tukey’s post hoc test (data in panel B were log_10_-transformed before analysis). *P* values <0.05 were considered statistically significant; ****P < 0.0001.

Groups of BALB/c laboratory mice (*N* = 8/group) were immunized with the same rS-WU1 or rS-Beta regimens mentioned above but at a 21-day interval. A negative control group (*N* = 5) was injected with vaccine formulation buffer. Spleens were harvested on day 28, seven days after the boost immunization (Fig. 5A). Splenocytes were collected and subjected to ELISpot and intracellular cytokine staining (ICS) to examine cytokine secretion upon stimulation with rS-WU1 or rS-Beta. ELISpot assay showed greater numbers of IFN-γ producing cells compared to the number of IL-5-producing cells upon all vaccination regimens, signifying a Th1-skewed response (Fig. 5B–D). Upon stimulation with either rS, strong Th1 responses were observed by ICS as measured by the presence of CD44^hi^CD62^low^CD4^+^ effector memory T cells expressing IFN-γ, IL-2, or TNF-α, and multifunctional CD4^+^ T cells expressing all three cytokines (Fig. 5E and Supplementary Fig. S2). Th2 CD4^+^ T cells that expressed IL-4 but were negative for IL-2 and TNF-α were also present, but at a lower proportion than that observed for Th1 cytokines (Supplementary Fig. S2). No significant differences in cytokine-positive cell numbers were observed among vaccination groups for any cytokine tested upon stimulation with either rS-WU1 or rS-Beta.

T-follicular helper cells (CXCR5^+^PD-1^+^CD4^+^) represent a group of CD4^+^ T cells in the lymphoid organs that aid germinal center formation and B cell development. We observed an increase in T-follicular helper cells in all vaccinated groups, though no statistically significant elevation was observed compared to placebo animals (Fig. 5F). Similarly, germinal center formation was evaluated by determining the percentage of GL7^+^CD95^+^CD19^+^ germinal center B cells among total B cells using flow cytometry. Although a tendency toward a higher percentage of germinal center B cells was observed in vaccinated groups compared to the placebo group, only animals immunized with the monovalent rS-Beta regimen showed a significantly higher proportion (*P* = 0.049 compared to placebo; Fig. 5G).

### Broad neutralization capacity of spike nanoparticle-based vaccines in BALB/c laboratory mice

**Fig. 6:**
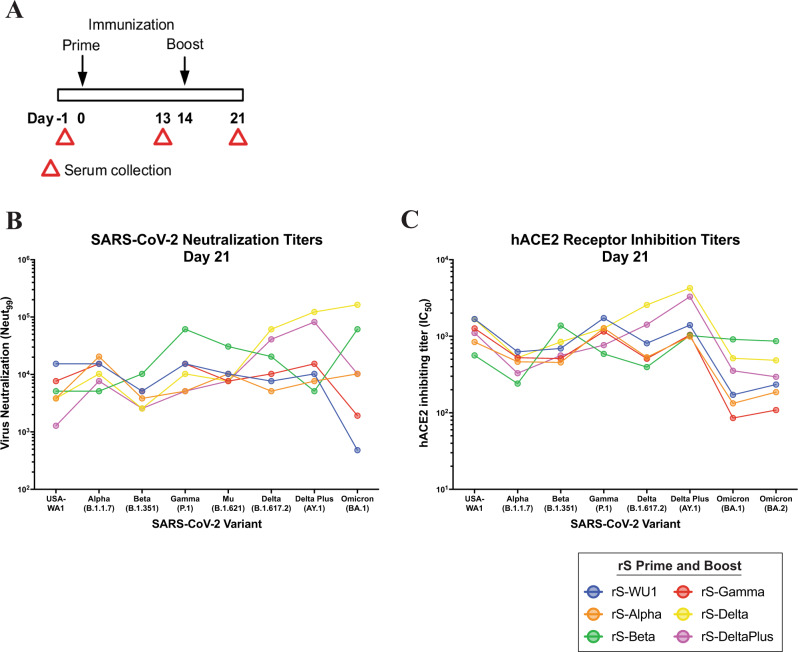
Cross-neutralizing antibody responses to spike nanoparticle-based vaccines in BALB/c laboratory mice. **A** Groups of mice (*N* = 20/group) were immunized in a prime/boost regimen on days 0 and 14 with 1 µg of SARS-CoV-2 rS based on Wuhan-Hu-1 (WU1), Beta, Alpha, Gamma, Delta, or Delta Plus rS with 5 μg of Matrix-M adjuvant. Sera were collected at the timepoints listed in the diagram. **B** SARS-CoV-2 neutralization antibody titers in sera collected on day 21 from *n* = 20 animals/group were determined using a CPE assay. Sera were evaluated for their ability to neutralize SARS-CoV-2 USA-WA1, Alpha, Beta, Gamma, Mu, Delta, Delta Plus, or Omicron BA.1 variants (*n* = 2 per sample). Data points indicate pooled serum titer from each group and connect lines connecting similar vaccines. **C** Functional antibody titers capable of disrupting binding between the SARS-CoV-2 receptor hACE2 and Wuhan-Hu-1, Alpha, Beta, Gamma, Delta, Delta Plus, Omicron BA.1 spike proteins listed in the figure were measured in sera collected on day 21. Data points indicate geometric mean titer from each group (*n* = 20/group).

The rapid and continuous emergence of SARS-CoV-2 variants has demonstrated the need for vaccines with broad neutralizing capability. We analyzed the broad neutralizing capacity of variant spike nanoparticle vaccines formulated with Matrix-M. In the first experiment, spike nanoparticles with spike sequences from Wuhan-Hu-1 (same as in the prototype vaccine NVX-CoV2373), B.1.1.7 (Alpha), B.1.351 (Beta), P.1 (Gamma), B.1.617.2 (Delta) and A.Y.4.1 (Delta Plus) were formulated with Matrix-M and used as individual spike vaccines. Mice were vaccinated and then boosted 14 days later before serum collection at day 21 (Fig. 6A). Sera from animals immunized with variant vaccines were evaluated for neutralization capacity across a panel of variants, including BA.1 (Fig. 6B and Supplementary Fig. S4). We found that for serum from animals immunized with rS-WU1, there was equal neutralization capacity across variants, except for a threefold reduction in neutralizing capacity against Beta, and over 30-fold reduction in neutralizing capacity against Omicron BA.1. The Beta spike vaccine demonstrated a broadest neutralizing capacity with high neutralization against the Beta variant but also very high neutralization against Gamma, Delta, Delta Plus, and Omicron BA.1. rS-Delta vaccination also showed broad neutralization coverage, including against Omicron BA.1 (Fig. 6B and Supplementary Fig. S4). Supporting these findings are assays testing the ability of the same serum to inhibit spike:hACE2 binding (Fig. 6C and Supplementary Fig. S5). Similar to the neutralizing antibody analysis, we found that serum from rS-WU1 vaccinated mice showed high inhibition of rS-WU1:ACE2 binding with reduced rS-Beta:hACE2 inhibition, correlating with neutralizing antibody titers. When sera from mice immunized with rS-Beta were tested, we observed broad spike:hACE2 inhibition of a rS-Beta spike vaccine, including against Omicron. These data correlate with the broad neutralization data showing similarly strong neutralization of Gamma, Delta, and Omicron BA.1 spike interactions. This demonstrates that adjuvanted spike nanoparticle vaccines can elicit broad protection against future variants.

## Discussion

As SARS-CoV-2 has spread, it has created selective pressure for viruses with increased fitness across multiple parameters, including replication rate, receptor binding, and antibody evasion^17,18^. This has produced several variant mutants that have gained or are gaining prevalence around the world. One of these variants, Beta, was first identified in South Africa^10^. This variant gained the ability to evade antibodies produced during both convalescence and vaccination and significantly evade currently authorized monoclonal antibody therapies. Based on the potential need for a spike variant-directed vaccine, Novavax produced a recombinant SARS-CoV-2 spike protein antigen based on the Beta lineage (rS-Beta). We have shown that a full-length, stabilized prefusion SARS-CoV-2 spike glycoprotein vaccine using the Beta variant spike adjuvanted by Matrix-M can induce high levels of functional immunity and protects mice against both Alpha and Beta SARS-CoV-2 variants. Immunizing Balb/c laboratory mice or boosting olive baboons with rS-Beta resulted in the production of anti-spike antibodies, hACE2-receptor inhibiting antibodies, and SARS-CoV-2 neutralizing antibodies. In addition, the rS-Beta vaccine induced Ag-specific CD4^+^ T-cell responses, induced germinal center formation and provided protection against in vivo challenge.

In mice, the antibodies produced after vaccination with the Beta variant-directed vaccine were able to inhibit binding between hACE2 and newly emergent variant spikes (e.g., Delta, Omicron) or ancestral spike to the same degree, indicating that this variant-directed vaccine could efficiently protect “backward” against ancestral SARS-CoV-2 variants and “forward” against newly emerging variants. In contrast, the prototype vaccine was less efficient at protecting “forward” against the Beta and Omicron variants. This finding corroborates previous findings that emerging strains of the human coronavirus 229E can evade antibody immunity^19^, enforcing the need for variant-directed SARS-CoV-2 vaccines during primary immunization or as booster vaccines. Though our mouse data did not demonstrate an added protection benefit to a bivalent vaccination or heterologous boosting strategy, our data does show a broadening of neutralizing antibody protection as compared to prototype vaccine. The nonhuman primate results presented in this work suggest that one dose of a Beta variant-directed vaccine may be sufficient for boosting regimens after previous immunization with a COVID-19 vaccine based on ancestral spike strains; two variant vaccine boosters did not seem to confer any added benefit over a single booster. In addition, a Beta-directed vaccine appears to broaden a vaccine response to other newly emerged variants, namely Delta and Omicron. Production of high neutralizing antibody titers after initial convalescence or after single vaccination points to the affinity maturation that occurs after first exposure to a new antigen. This immunological training may be essential for durability.

The vaccine dosing strategy used in this study was designed to be consistent with previously published mouse and nonhuman primate testing of protein subunit vaccines. In general, protein-based vaccines require an adjuvant to stimulate antigenicity. In mouse studies, the 1 µg/mouse used here is similar to that used in additional studies of protein-based vaccines^16,20–30^. It should be noted that the doses used for our mouse and baboon studies are higher when normalized by weight than what has now been authorized by the United States Food and Drug Administration for use in humans (5 µg protein, 50 µg adjuvant). We do not believe that reductions in vaccine amount per dose in our experiments would not have substantial effects on neutralization breadth. Across these studies cited above, the immunogenicity varies little compared to dose. As we have observed in mRNA studies, lowering the dose of vaccine 10–50-fold has little effect on broad protection and only on magnitude of antibody present^31^. However, the protection from disease and viral replication demonstrated in this mouse model in combination with the broadening neutralization demonstrated in our rS-Beta boosted baboon data suggests a benefit to an rS-Beta-directed or bivalent booster in humans to broaden a neutralizing antibody response to future variants.

The emergence of variants and potential for changes in vaccine composition throughout the COVID-19 pandemic is much more rapid than the current schedule for Influenza vaccines. Multiple criteria impact the choice on whether to develop specific variant-directed vaccines, including not only loss of neutralization and protection from current circulating strains, but also distribution, infrastructure, booster uptake and disease model demonstration of protection against future emerging strains. As shown in our manuscript, a rS-WU1/rS-Beta vaccine provides broad coverage across lineages through BA.1. The ideal vaccine would be highly immunogenic, protect against current and near-current variants as well as produce immune memory for recall responses that would protect against divergent strains that have not emerged yet. One way to do this is to evaluate the recognition and neutralization of current strains by Spike vaccines alone or in combination, as we have done here. We believe that combinations of Beta or future Spike variants with a WU1 spike allow for the best protection in this protein-based recombinant spike vaccine. Vaccination strategies with Beta Spike have also been recently published in NHPs with an mRNA vaccine and hamsters with a ChAdOx1-based vaccine^32–34^. In these studies, it is demonstrated that Beta Spike-containing vaccines are highly immunogenic and offer broader protection against variants. Interestingly, the vaccine platform can also affect neutralization breadth and the vaccination status of the individual can certainly have an impact on how responses will be modulated in an individual system^35–37^. As shown in this study, the use of an adjuvant in the Novavax vaccines certainly impacts the immunogenicity of the Spike protein as well as antigen presentation, as seen in other adjuvant:antigen combinations^38–41^. The Matrix M1 adjuvant used in NVX-2373 may provide enhanced neutralization and protection across variants as compared to other vaccine platforms. We are currently focused on these mechanistic parameters as we compare Spike vaccines across multiple models.

There are strengths and weaknesses to the work described in this manuscript. The strengths are the broad neutralization identified for the Beta and Delta variant Spikes used as vaccine antigens and the consistent immune response seen in both mice and baboons in their response to beta vaccination. As noted, the doses used in mice and baboons are consistent with other published protein-based models however they are higher than would be seen in humans in a weight/dose comparison. The weaknesses surround the pathogenesis experiments where Alpha and Beta variant infections are compared in mice. Since Alpha has a lower peak virus titer in mice, even though it has a 501Y Spike mutation, the differential between titer and clearance is smaller than compared to the Beta variant. This limits the power of the Alpha variant infection however neutralization data to Alpha corresponds with our in vivo findings.

As variant viruses continue to emerge across the world, they are being selected for in the background of increasing seroprevalence. Vaccines must be able to produce high antibody titers across a wide range of age groups and comorbidities, and the vaccine platform must be able to rapidly respond to the variants at the earliest signs of their emergence. The baculovirus-Sf9 cell core technology used to produce rS protein is highly amenable to a rapid response to emergent SARS-CoV-2 variants of concern; producing a variant-directed recombinant spike antigen requires few to no changes in the purification and manufacturing processes developed for producing prototype spike vaccines. In addition, this vaccine technology does not utilize human fetal or embryonic cells during vaccine testing or manufacturing, nor are human fetal or embryonic cells or tissue contained in the vaccine. After a prototype vaccine is shown to meet critical immunogenicity, efficacy, and safety benchmarks, variant-directed vaccines can also reach the clinic on an accelerated timeline from a regulatory standpoint.

A booster vaccination containing a single or multiple variant rS vaccines could both increase antibody levels as well as broaden coverage to variants (e.g., Delta, Omicron), as shown in this work. Others have shown additional experiments with other combinations of Spike proteins in protein or mRNA-based vaccines that also provide protection for a range of variants. After our work was completed, Omicron variants emerged from a wholly separate lineage of SARS-CoV-2^35–37^. Vaccination with Omicron BA.1 Spike proteins, across a range of platforms demonstrates strong Omicron neutralization but little prototype neutralization. This shows the broad difficulty in picking strains for single or combination vaccines to protect from future unknown variants that will emerge. We demonstrated that Beta and Delta vaccinated mouse sera neutralize a wide range of variants, including Omicron BA.1. When combination vaccines of WU1 and Beta were used, we observed broad protection against Alpha and Beta but also high neutralizing antibody titers against newly emerged variants of concern such as Delta and Omicron. This demonstrates the high level of protective effects, via neutralizing antibody levels, that heterologous adjuvanted protein-based vaccines can have for current variants as well as those yet to emerge.

## Methods

### Cells and virus

Virus and cells were processed as described previously^16^. Briefly, VeroE6 cells (ATCC# CRL 1586) or VeroE6 cells overexpressing TMPRSS2 were cultured in DMEM (Quality Biological), supplemented with 10% (v/v) fetal bovine serum (Gibco), 1% (v/v) penicillin/streptomycin (Gemini Bio-products) and 1% (v/v) L-glutamine (2 mM final concentration, Gibco) (Vero media). Cells were maintained at 37 °C and 5% CO_2_. SARS-CoV-2/Alpha (B.1.17) (hCoV-19/USA/MD-HP01101/2021) and SARS-CoV-2/Beta (B.1.351) (hCoV-19/USA/MD-HP01542/2021) were generously provided by Dr. Andy Pekosz at The Johns Hopkins University. SARS-CoV-2/Gamma (P.1) hCoV-19/Japan/TY7-503/2021, SARS-CoV-2/Delta (B.1.617.2) hCoV-19/USA/MD-HP05285/2021, and SARS-CoV-2/Omicron (BA.1) hCoV-19/USA/GA-EHC-2811C/2021 were provided by BEI. Stocks for both viruses were prepared by infection of Vero/TMPRSS2 cells for two days when CPE was starting to be visible^42^. Media were collected and clarified by centrifugation prior to being aliquoted for storage at −80 °C. Titer of stock was determined by plaque assay using VeroE6 cells as described previously^43^. All work with infectious virus was performed in a Biosafety Level 3 laboratory and approved by our Institutional Biosafety Committee.

### SARS-CoV-2 protein expression

SARS-CoV-2 constructs were synthetically produced from the full-length S glycoprotein gene sequence (GenBank MN908947 nucleotides 21563-25384). The full-length *S-*genes were codon optimized for expression in *Spodoptera frugiperda* (Sf9) cells and synthetically produced by GenScript® service (GenScript USA, Piscataway, NJ, USA). The QuikChange® Lightning site-directed mutagenesis kit (Agilent Technologies, Inc.) was used to produce two spike protein variants: the furin cleavage site (682-RRAR-685) was mutated to 682-QQAQ-685 to be protease resistant and two proline substitutions at positions K986P and V987P (2 P) were introduced to produce the double mutant, BV2373. To generate the recombinant spike constructs based on the Alpha B.1.1.7, Beta B.1.351, Gamma P.1, Delta B.1.617.2, and Delta Plus AY1 variants, additional variant-specific point mutations were introduced to BV2373 as indicated in Table 2 and Supplementary Fig. S3. Full-length *S*-genes were cloned between the BamHI–HindIII sites in the pFastBac baculovirus transfer vector (Invitrogen, Carlsbad, CA) under transcriptional control of the *Autographa californica* polyhedron promoter. Recombinant baculovirus constructs were plaque purified and master seed stocks prepared and used to produce the working virus stocks. The baculovirus master and working stock titers were determined using rapid titer kit (Clontech, Mountain View, CA). Recombinant baculovirus stocks were prepared by infecting Sf9 cells with a multiplicity of infection (MOI) of ≤0.01 plaque forming units (pfu) per cell.

**Table 2:** Variant SARS-CoV-2 recombinant spike protein constructs

Vaccine construct	Accession #	Mutations from NVX-CoV2373 rS
SARS-CoV-2 Alpha rS (BV2425)	GISAID EPI_ISL_683466	Δ69-70, Δ144, N501Y, A570D, D614G, P681H, T716I, S982A, D1118H
SARS-CoV-2 Beta rS (BV2438)	GISAID EPI_ISL_696502	D80A, D215G, L242H, K417N, E484K, N501Y, D614G, A701V
SARS-CoV-2 Gamma rS(BV2443)	GISAID EPI_ISL_833174	L18F, T20N, P26S, D138Y, R190S, K417T, E484K, N501Y, D614G, H655Y, T1027I, V1176F
SARS-CoV-2 Delta rS(BV2465)	GISAID EPI_ISL_2133949	T19R, G142D, Δ156, Δ157, R158G, L452R, T478K, D614G, P681R, D950N
SARS-CoV-2 Delta Plus rS(BV2472)	GISAID EPI_ISL_2439552	T19R, G142D, R158G, Δ156, Δ157, W258I, K417N, L452R, T478K, D614G, P681R, D950N

### Expression and purification

SARS-CoV-2 rS proteins were produced in Sf9 cells as previously described^16^. Briefly, cells were expanded in a serum-free medium and infected with recombinant baculovirus. Cells were cultured at 27 ± 2 °C and harvested at 68−72 h post infection by centrifugation (4000×*g* for 15 min). Cell pellets were suspended in 25 mM Tris HCl (pH 8.0), 50 mM NaCl and 0.5–1.0% (v/v) TERGITOL NP-9 with leupeptin. rS proteins were extracted from the plasma membranes with Tris buffer containing NP-9 detergent, clarified by centrifugation at 10,000×*g* for 30 min. rS proteins were purified by TMAE anion exchange and lentil lectin affinity chromatography. Hollow fiber tangential flow filtration was used to formulate the purified spike protein at 100–150 μg mL^−1^ in 25 mM sodium phosphate (pH 7.2), 300 mM NaCl, 0.02% (v/v) polysorbate 80 (PS-80). Purified rS proteins were evaluated by 4–12% gradient SDS-PAGE stained with SimplyBlue SafeStain Coomassie reagent (ThermoFisher Scientific, Waltham, MA) and purity was determined by scanning densitometry using the Image Lab system (BioRad, Hercules, CA).

### Differential scanning calorimetry (DSC)

Samples (BV2426 Lot 01Feb21 and BV2373 Lot 15Dec20; rS-Beta and rS-WU1, respectively) and corresponding buffers were heated from 0 °C to 100 °C at 1 °C per minute and the differential heat capacity change was measured in a NanoDSC (TA Instruments, New Castle, DE). A separate buffer scan was performed to obtain a baseline, which was subtracted from the sample scan to produce a baseline-corrected profile. The temperature where the peak apex is located is the transition temperature (*T*_max_) and the area under the peak provides the enthalpy of transition (Δhcal).

### Transmission electron microscopy (TEM) and 2D class averaging

Electron microscopy was performed by NanoImaging Services (San Diego, CA) with a ThermoFisher Scientific Glacios Cryo Transmission Electron Microscope, operated at 200 kV equipped with a Falcon 3 direct electron detector. SARS-CoV-2 rS proteins were diluted to 16 µg mL^−1^ in formulation buffer. The samples (3 µL) were applied to continuous carbon grids and stained with uranyl format. Images of each grid were acquired at multiple scales to assess the overall distribution of the sample. High-magnification images were acquired at nominal magnifications of 150,000× (0.095 nm/pixel) and 92,000× (0.159 nm/pixel). The images were acquired at a nominal defocus of −2.0 µm to −1.5 µm and electron doses of ~25 e^−^/Å^2^.

For class averaging, particles were identified from ×92,000 high-magnification images, followed by alignment and classification as previously described^16^.

### Kinetics of SARS-CoV-2 S binding to hACE2 receptor by BLI

rS protein receptor binding kinetics was determined by bio-layer interferometry (BLI) using an Octet QK384 system (Pall Forté Bio, Fremont, CA). His-tagged human ACE2 (2 μg mL^−1^) was immobilized on nickel-charged Ni-NTA biosensor tips. After baseline, SARS-CoV-2 rS protein solutions were twofold serially diluted in kinetics buffer over a range of 300 nM to 4.7 nM, allowed to associate for 600 s, followed by dissociation for an additional 600–900 s. Data were analyzed with Octet software HT 10.0 by 1:1 global curve fit.

### Animal ethics statement

The mouse immunizations were performed by Noble Life Sciences (Sykesville, MD) before transfer to UMSOM BSL3 facility for challenge. Noble Life Sciences and the University of Maryland School of Medicine are accredited by the Association for Assessment and Accreditation of Laboratory Animal Care (AAALACC International). All animal procedures were in accordance with NRC Guide for the Care and Use of Laboratory Animals, the Animal Welfare Act, and the CDC/NIH Biosafety in Microbiological and Biomedical Laboratories. Mouse challenge studies were approved by The University of Maryland School of Medicine IACUC. The olive baboon (*Papio cynocephalus nubis*) study was performed at the University of Oklahoma Health Science Center (OUHSC). OUHSC is accredited by AAALACC International. Baboons were maintained and treated according to the Institutional Biosafety Committee guidelines. Baboon experiments were approved by the Institutional Animal Care and Use Committee (IACUC) and the Institutional Biosafety Committee of OUHSC. Studies were conducted in accordance with the National Institutes of Health Guide for Care and Use of Laboratory Animals (NIH publication 8023, Revised 1978).

### Mouse study designs

Female BALB/c laboratory mice (7–9 weeks old, 17–22 g, *N* = 20 per group) were immunized by intramuscular (IM) injection with two doses spaced 14 days apart (study day 0 and 14) of rS-WU1, rS-Beta with 5 μg saponin-based Matrix-M™ adjuvant (Novavax, AB, Uppsala, SE) either alone, in combination, or as a heterologous prime/boost. Vaccine dosing was consistent with previously published mouse testing of our rS-WU1 vaccine^16,27^. A placebo group was injected with vaccine formulation buffer as a negative control. Serum was collected for analysis on study days −1, 14, 21, and 32. Vaccinated and control animals were intranasally challenged with SARS-CoV-2 on study day 46.

To assess the cellular response mediated by Matrix-M adjuvant, groups of female BALB/c mice (*N* = 8 per group) were immunized IM with the same regimens described above, with injections spaced 21 days apart. Spleens were collected 7 days after the second immunization (study day 28). A non-vaccinated group (*N* = 5) served as a control.

To assess cross-neutralizing antibody responses, female BALB/c mice (*N* = 20 per group) were immunized by intramuscular (IM) injection with two doses spaced 14 days apart (study day 0 and 14) of rS-WU1, rS-Alpha (B.1.17), rS-Beta, rS-Gamma (P.1), rS-Delta (B.1.617.2), or rS-Delta Plus with 5 μg Matrix-M adjuvant as homologous prime/boost. Serum was collected for analysis on study days −1, 14, and 21. Information about spike protein antigens used for immunization can be found in Supplementary Fig. S3.

### Baboon study design

Nine adult baboons (10–16 years of age at study initiation) were randomized into 4 groups of 2–3/group and immunized by IM injection with rS-WU1 at 1, 5, or 25 μg rS with 50 μg Matrix-M adjuvant. A separate group was immunized with 25 μg rS without adjuvant (Supplementary Table S1). Animals were vaccinated with 2 doses spaced 21 days apart in this primary immunization series. Immunogenicity results after the primary immunization series were previously described^16^. Approximately 1 year later (45 weeks), all animals were boosted with one or two 3 μg doses of rS-Beta with 50 μg Matrix-M adjuvant. Sera and PBMCs were collected before and after the boost to measure antibody- and cell-mediated immune responses.

### SARS-CoV-2 challenge in mice

Mice were anaesthetized by intraperitoneal injection 50 μL of a mix of xylazine (0.38 mg/mouse) and ketamine (1.3 mg/mouse) diluted in phosphate-buffered saline (PBS). Mice were intranasally inoculated with either 7 × 10^4^ pfu of Alpha or 1 × 10^5^ pfu of Beta variants of SARS-CoV-2 in 50 μL. Challenged mice were weighed on day of infection and daily for 4 days post infection. At days 2 and 4 post infection, five mice were sacrificed from each vaccination and control group, and lungs were harvested to determine viral titer by a plaque assay and viral RNA levels by qRT-PCR.

### SARS-CoV-2 plaque assay

SARS-CoV-2 plaque assays were performed as previously described^16^. Assays were performed for all samples of each group (*n* = 5–10 per group).

### SARS-CoV-2 subgenomic RNA detection

Lung tissue was harvested and homogenized in TRIzol (Ambion). RNA was extracted per the manufacturer’s instructions using the Direct-zol RNA Miniprep Kit (Zymo Research). RNA was converted into cDNA (RevertAid Reverse Transcriptase) and used as template for qPCR (Applied Biosystems PowerUp SYBR Green Master Mix, Cat #A25742). The primers used were against the N gene (5′-TAATCAGACAAGGAACTGATTA-3′ (forward) and 5′-CGAAGGTGTGACTTCCATG-3′ (reverse)) on an Applied Biosystems QuantStudio 5 thermocycler (*n* = 5–10 per group). Data were analyzed in Prism 9 (Graphpad).

### Anti-SARS-CoV-2 spike IgG by ELISA

An ELISA was used to determine anti-SARS-CoV-2 S IgG titers. Briefly, 96-well microtiter plates (ThermoFischer Scientific, Rochester, NY, USA) were coated with 1.0 µg mL^−1^ of SARS-CoV-2 spike protein. Plates were washed with PBS-T and blocked with TBS Startblock blocking buffer (ThermoFisher, Scientific). Mouse and baboon serum samples were serially diluted (10^−2^ to 10^−8^) and added to the blocked plates before incubation at room temperature for 2 h. Following incubation, plates were washed with PBS-T and HRP-conjugated goat anti-mouse IgG or goat anti-human IgG (Southern Biotech, Birmingham, AL, USA) added for 1 h. Plates were washed with PBS-T and 3,3’,5,5’-tetramethylbenzidine peroxidase substrate (TMB, T0440-IL, Sigma, St Louis, MO, USA) was added. Reactions were stopped with TMB stop solution (ScyTek Laboratories, Inc. Logan, UT). Plates were read at OD 450 nm with a SpectraMax®Plus plate reader (Molecular Devices, Sunnyvale, CA, USA), and data were analyzed with SoftMax® (Molecular Devices, Corp.) software. Data shown in the graph are the average of triplicate wells. EC_50_ values were calculated by 4-parameter fitting using SoftMax Pro 6.5.1 GxP software. Individual animal anti-SARS-CoV-2 rS IgG titers and group geometric mean titers (GMT) and 95% confidence interval (± 95% CI) were plotted GraphPad® Prism 7.05 software (Graphpad Software LLC).

### hACE2-receptor-blocking antibodies

Human ACE2 receptor-blocking antibodies were determined by ELISA. Ninety-six well plates were coated with 1.0 μg mL^−1^ SARS-CoV-2 rS protein overnight at 4 °C. After washing with PBS-T and blocking with StartingBlock (TBS) blocking buffer (ThermoFisher Scientific), serially diluted serum from groups of immunized mice or baboons were added to coated wells and incubated for 1 h at room temperature. After washing, 30 ng mL^−1^ of histidine-tagged hACE2 (Sino Biologics, Beijing, CHN) was added to wells for 1 h at room temperature. After washing, HRP-conjugated anti-histidine IgG (Southern Biotech, Birmingham, AL, USA) was added, followed by washing and the addition of TMB substrate. Plates were read at OD 450 nm with a SpectraMax plus plate reader (Molecular Devices, Sunnyvale, CA, USA), and data were analyzed with SoftMax Pro 6.5.1 GxP software. Data shown in the graph are the average of triplicate wells. The % Inhibition for each dilution for each sample was calculated using the following equation in the SoftMax Pro program: 100 − [(MeanResults/ControlValue@PositiveControl)*100].

Serum dilution versus % Inhibition plot was generated and curve fitting was performed by four parameter logistic (4PL) curve fitting to data. Serum antibody titer at 50% inhibition (IC_50_) of hACE2 to SARS-CoV-2 rS protein (rS-WU1 or rS-Beta) was determined in the SoftMax Pro program.

### SARS-CoV-2 neutralization titer by plaque reduction neutralization titer assay (PRNT)

PRNTs were processed as described previously^44^. Briefly, serum samples were diluted in DMEM (Quality Biological) at an initial 1:40 (baboon or mouse samples) dilution with 1:2 serial dilutions for a total of 11 dilutions. A no-sera control was included on every plate. SARS-CoV-2 (WA1, B1.1.7, or B1.351) was then added 1:1 to each dilution for a target of 50 PFU per plaque assay well and incubated at 37 °C (5.0% CO_2_) for 1 h. Sample titers where then determined by plaque assay, and neutralization titers determined as compared to the non-treatment control. Data shown in the graph are the average of duplicate wells. A 4-parameter logistic curve was fit to these neutralization data in PRISM (GraphPad, San Diego, CA), and the dilution required to neutralize 50% of the virus (PRNT50) was calculated based on that curve fit.

### SARS-CoV-2 neutralization titer by microneutralization assay (Neut99)

SARS-CoV-2 microneutralization was processed as described previously^16^. Briefly, serum samples were diluted as described for the PRNT assay. SARS-CoV-2 inoculum was then added to result in an MOI of 0.01 pfu per cell and incubated for 60 min at 37 °C before adding the mixture to VeroE6 cells overexpressing TMPRSS2. CPE was assessed after 3 days, with the first well to show CPE recorded as the endpoint neutralization titer.

### Surface and intracellular cytokine staining

For surface staining, murine splenocytes were first incubated with an anti-CD16/32 antibody to block the Fc receptor. To characterize T-follicular helper cells (Tfh), splenocytes were incubated with the following antibodies or dye: BV650-conjugated anti-CD3, APC-H7-conjugated anti-CD4, FITC-conjugated anti-CD8, Percp-cy5.5-conjugated anti-CXCR5, APC-conjugated anti-PD-1, Alexa Fluor 700-conjugated anti-CD19, PE-conjugated anti-CD49b (BD Biosciences, San Jose, CA) and the yellow LIVE/DEAD® dye (Life Technologies, NY). To stain germinal center (GC) B cells, splenocytes were labeled with FITC-conjugated anti-CD3, PerCP-Cy5.5-conjugated anti-B220, APC-conjugated anti-CD19, PE-cy7-conjugated anti-CD95, and BV421-conjugated anti-GL7 (BD Biosciences) and the yellow LIVE/DEAD® dye (Life Technologies, NY).

For intracellular cytokine staining (ICCS) of murine splenocytes, cells were cultured in a 96-well U-bottom plate at 2 × 10^6^ cells per well. The cells were stimulated with rS-WU1 or rS-Beta spike protein. The plate was incubated 6 h at 37 °C in the presence of BD GolgiPlug™ and BD GolgiStop™ (BD Biosciences) for the last 4 h of incubation. Cells were labeled with murine antibodies against CD3 (BV650), CD4 (APC-H7), CD8 (FITC), CD44 (Alexa Fluor 700), and CD62L (PE) (BD Pharmingen, CA) and the yellow LIVE/DEAD® dye. After fixation with Cytofix/Cytoperm (BD Biosciences), cells were incubated with PerCP-Cy5.5-conjugated anti-IFN-γ, BV421-conjugated anti-IL-2, PE-Cy7-conjugated anti-TNF-α, and APC-conjugated anti-IL-4 (BD Biosciences). All stained samples were acquired using a LSR-Fortessa or a FACSymphony flow cytometer (Becton Dickinson, San Jose, CA) and the data were analyzed with FlowJo software version Xv10 (Tree Star Inc., Ashland, OR). For ICS of baboon PBMCs, PBMCs collected at the timepoints listed in Fig. 5A were stimulated as described above with rS-WU1 or rS-Beta. Cells were labeled with human/NHP antibodies BV650-conjugated anti-CD3, APC-H7-conjugated anti-CD4, FITC-conjugated anti-CD8, BV421-conjugated anti-IL-2, PerCP-Cy5.5-conjugated anti-IFN-γ, PE-Cy7-conjugated anti-TNF-α, APC-conjugated anti-IL-5, BV711-conjugated anti-IL-13 (BD Biosciences), and the yellow LIVE/DEAD® dye.

### ELISpot assay

Murine IFN-γ and IL-5 ELISpot assays were performed following the manufacturer’s procedures for mouse IFN-γ and IL-5 ELISpot kits (3321-2H and 3321-2 A, Mabtech, Cincinnati, OH). Briefly, 4 × 10^5^ splenocytes in a volume of 200 µL were stimulated with rS-WU1 or rS-Beta in plates that were pre-coated with anti-IFN-γ or anti-IL-5 antibodies. Each stimulation condition was carried out in triplicate. Assay plates were incubated 24–48 h at 37 °C in a 5% CO_2_ incubator and developed using BD ELISpot AEC substrate set (BD Biosciences, San Diego, CA). Spots were counted and analyzed using an ELISpot reader and ImmunoSpot®software v6 (Cellular Technology, Ltd., Shaker Heights, OH). The number of IFN-γ- or IL-5-secreting cells was obtained by subtracting the background number in the medium controls. Data shown in the graph are the average of triplicate wells.

Similarly, baboon IFN-γ and IL-4 assays were carried out using NHP IFN-γ and Human IL-4 assay kits from Mabtech. Assays were performed in triplicate.

### Statistical analysis

Statistical analyses were performed with GraphPad Prism 9.0 software (La Jolla, CA). Serum antibody titers were plotted for individual animals and the geometric mean titer (GMT) and 95% confidence interval (95% CI) or the means ± SEM as indicated in the figure. Ordinary one-way ANOVA with Dunnett comparisons post hoc test was performed on log_10_-transformed data to evaluate the statistical significance of differences among groups. *P* values ≤0.05 were considered as statistically significant.

### Reporting summary

Further information on research design is available in the Nature Portfolio Reporting Summary linked to this article.

## Supplementary information

Supplementary Information

Reporting Summary

## Data Availability

The authors declare that all data supporting the findings of the study are available in the article and the Supplementary Information file. Source data are provided with this paper.

## Source data

Source Data
