# Supplementary material for: Immunogenicity and protection of a variant nanoparticle vaccine that confers broad neutralization against SARS-CoV-2 variants
Source: Nat Commun. 2023 Feb 28;14:1130. doi: 10.1038/s41467-022-35606-6 (PMC9972327; doi:10.1038/s41467-022-35606-6)
Supplement: Supplementary file 1 — Supplementary Information [file 41467_2022_35606_MOESM1_ESM.pdf]

**Title**: **Immunogenicity and In vivo protection of a variant nanoparticle vaccine that confers broad protection against emerging SARS-CoV-2 variants**

**Authors:** James Logue[1]*, Robert M. Johnson[1]*, Nita Patel[2], Bin Zhou[2], Sonia Maciejewski[2], Haixia Zhou[2], Alyse D. Portnoff[2], Jing-Hui Tian[2], Asma Rehman[2], Marisa E. McGrath[1], Robert E. Haupt[1], Stuart M. Weston[1], Lauren Baracco[1], Holly Hammond[1], Mimi Guebre-Xabier[2], Carly Dillen[1], , M Madhangi[2], Ann M Greene[2], Michael J. Massare[2], Greg M Glenn[2], Gale Smith[2], Matthew B. Frieman[1#]

[1] The Department of Microbiology and Immunology, The University of Maryland School of Medicine, Baltimore, MD, 21201.

[2] Novavax, Inc. 21 Firstfield Road, Gaithersburg, MD 20878.

#Corresponding Author: mfrieman@som.umaryland.edu

*These authors contributed equally to this work.

**This PDF file includes:**

Table S1: Baboon Study Animal Information

Figure S1: Individual Cytokine responses to NVX-CoV2438 boost in Baboons

Figure S2: Individual Cytokine responses in Mice

Figure S3: Variant SARS-CoV-2 Recombinant Spike Protein Constructs

Figure S4: SARS-CoV-2 neutralization antibody titers

Figure S5: SARS-CoV-2 hACE2 receptor binding inhibition titers

Supplemental Material

Table S1

| Immunization Group | Animal ID# | Animal Weights at D0 (kg) | rS-WU1 (D0, D21) | rS-Beta Booster (D318) | (D339) |
|---|---|---|---|---|---|
| 1 | **804** | 34.2 | 25µg rS-WU1<br>0µg Matrix-M1 | 3µg rS-Beta<br>50µg Matrix-M1 | *N/A* |
| | **8809** | 14.8 | 25µg rS-WU1<br>0µg Matrix-M1 | 3µg rS-Beta<br>50µg Matrix-M1 | 3µg rS-WU1<br>50µg Matrix-M1 |
| 2 | **9206** | 30.4 | 1µg rS-WU1<br>50µg Matrix-M1 | 3µg rS-Beta<br>50µg Matrix-M1 | *N/A* |
| | **5909** | 17.9 | 1µg rS-WU1<br>50µg Matrix-M1 | 3µg rS-Beta<br>50µg Matrix-M1 | 3µg rS-WU1<br>50µg Matrix-M1 |
| 3 | **7207** | 43.6 | 5µg rS-WU1<br>50µg Matrix-M1 | 3µg rS-Beta<br>50µg Matrix-M1 | *N/A* |
| | **6103** | 21.4 | 5µg rS-WU1<br>50µg Matrix-M1 | 3µg rS-Beta<br>50µg Matrix-M1 | 3µg rS-WU1<br>50µg Matrix-M1 |
| 4 | **8006** | 30.6 | 25µg rS-WU1<br>50µg Matrix-M1 | 3µg rS-Beta<br>50µg Matrix-M1 | *N/A* |
| | **6904** | 17.4 | 25µg rS-WU1<br>50µg Matrix-M1 | 3µg rS-WU1<br>50µg Matrix-M1 | 3µg rS-WU1<br>50µg Matrix-M1 |
| | **1004** | 15.2 | 25µg rS-WU1<br>50µg Matrix-M1 | 3µg rS-WU1<br>50µg Matrix-M1 | 3µg rS-WU1<br>50µg Matrix-M1 |

Figure S1. Individual Cytokine responses to NVX-CoV2438 boost in Baboons

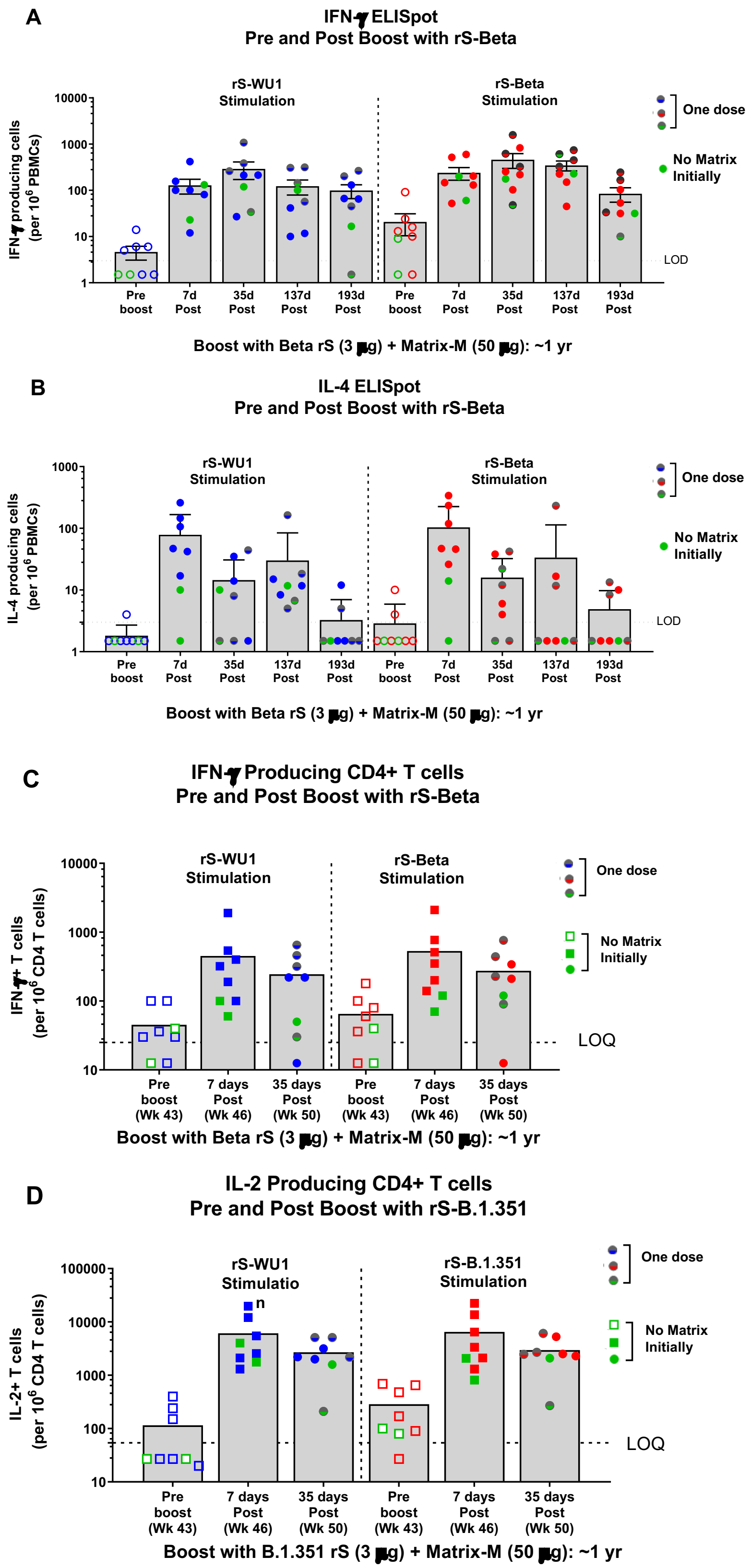

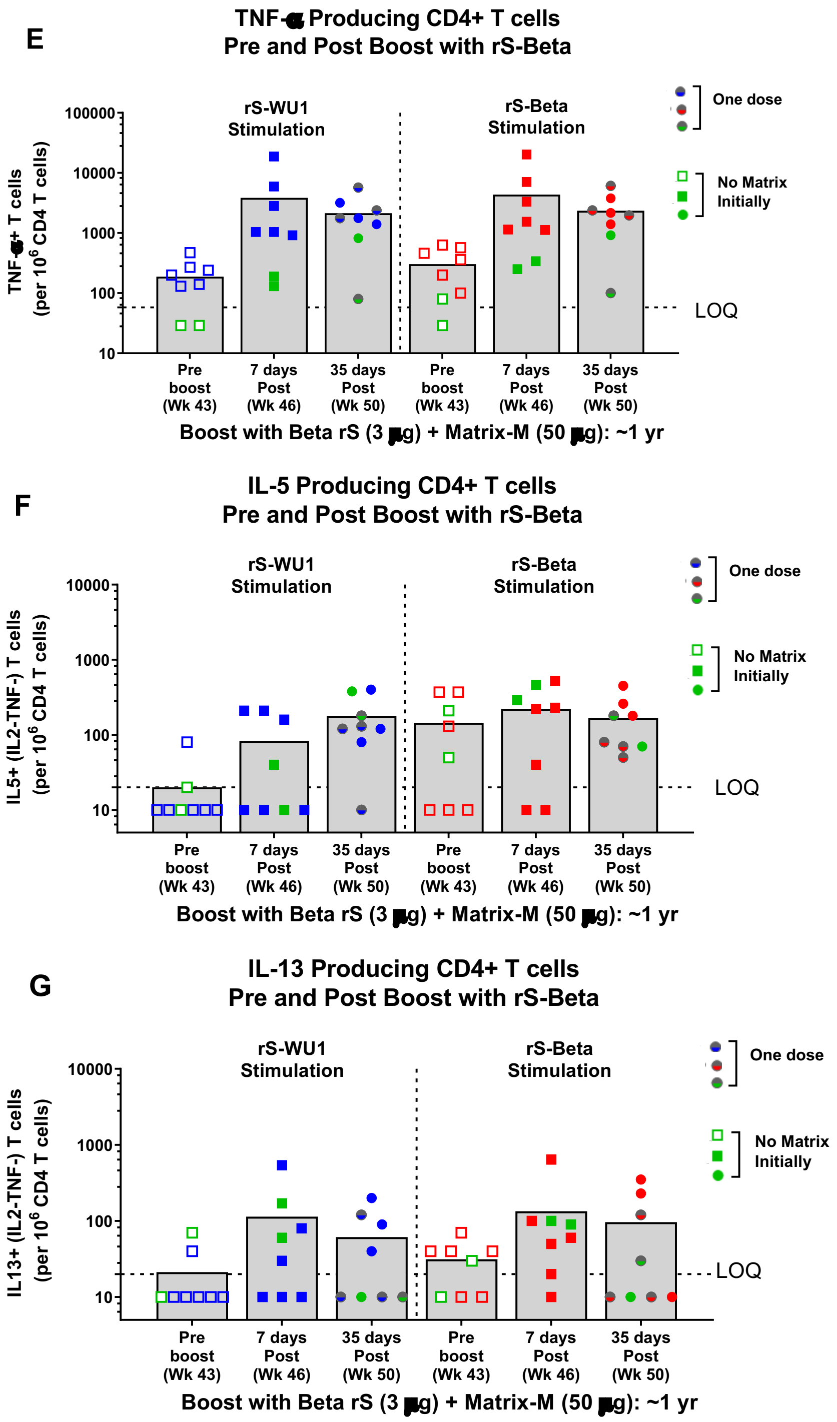


**Figure S1. Individual Cytokine Responses to rS-B.1.351 boost in Baboons.**

A small cohort of baboons (n = 2-3/group) was immunized with 1 µg, 5 µg, or 25 µg rS-WU1 with 50 µg Matrix-M1 adjuvant or unadjuvanted 25 µg rS-WU1 on Day 0 and 21 (Week 0 and 3, respectively). Approximately 1 year later, all animals were boosted with one or two doses of 3 µg rS-B.1.351 with 50 µg Matrix-M1 adjuvant on Day 318 and 339 (Weeks 45 and 48, respectively). PBMCs collected pre-boost (Day 303; Week 43), 7 days after the first rS-B.1.351

boost (Day 325; Week 46), 35 days after the first rS-B.1.351 boost (Day 353; Week 50), 137 days after the first rS-B.1.351 boost (Day 455; Week 65), and 193 days after the first rS-B.1.351 boost (Day 511; Week 73). PBMCs were stimulated with rS-WU1 or rS-B.1.351 and subjected to ELISpot assay to measure (**A**) IFN-γ producing cells as a Th1 cytokine and (**B**) IL-4 producing cells as a Th2 cytokine. CD4+ T cells were also stimulated with rS-WU1 or rS-B.1.351, then subjected to ICS to measure cells producing IFN-γ (**C**), IL-2 (**D**), TNF-α (**E**), IL-5 (**F**), and IL-13 (**G**).

Figure S2. Individual Cytokine responses in Mice

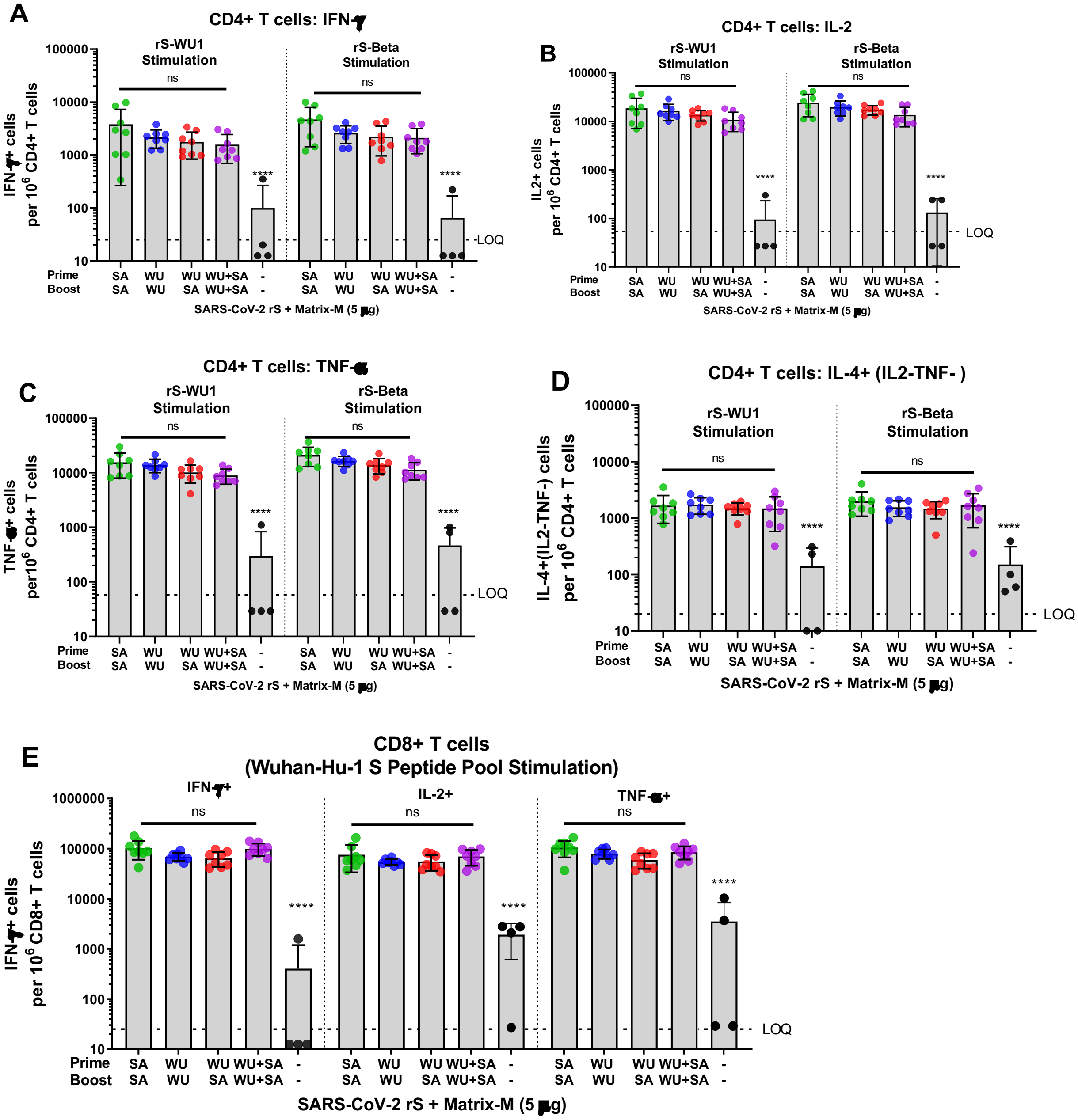


**Figure S2. Individual Cytokine responses in Mice**

Groups of mice (n = 8/group) were immunized in a prime/boost regimen on Days 0 and 21 with various combinations of SARS-CoV-2 rS based on B.1.351 (SA) or Wuhan-Hu-1 (WU). Mice were either primed and boosted with rS-B.1.351, primed and boosted with rS-WU1, primed with rS-WU1 and boosted with rS-B.1.351, or primed and boosted with bivalent rS-WU1+rS-B.1.351. Antigen doses were 1 µg rS for each monovalent immunization, or 1 µg rS for each construct upon bivalent immunization (2 µg rS total). All antigen doses were administered with 5 µg Matrix-M1 adjuvant. A control group received formulation buffer (Placebo, n = 5). Spleens were

harvested on Day 28 for cell collection. **A-D**. Isolated splenocytes were stimulated with either rS-WU1 or rS-B.1.351, then subjected to intracellular cytokine staining to determine whether CD4+ T cells were positive for IFN-γ (**A**), IL-2 (**B**), TNF-α (**C**), or IL-4 (**D**). **E.** To examine CD8+ T cell responses, cells were stimulated with a peptide pool corresponding to the entire Wuhan-Hu-1 spike protein sequence, then subjected to ICS for IFN-γ, IL-2, and TNF-α. Differences among experimental groups were evaluated by one-way ANOVA with Tukey's post-hoc test. P values $< 0.05$ were considered statistically significant; **** = $p < 0.0001$.

Figure S3. Variant SARS-CoV-2 Recombinant Spike Protein Constructs

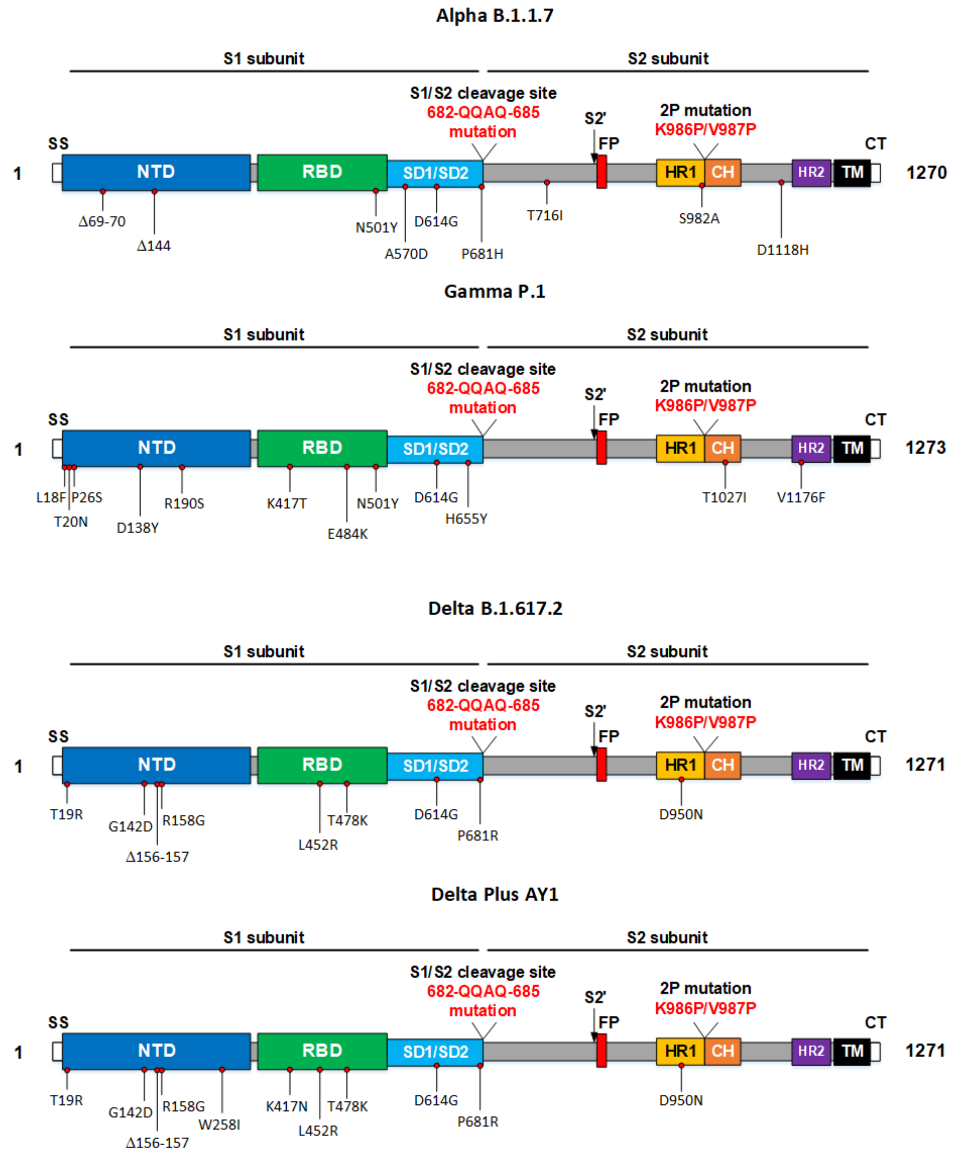


**Figure S3. Variant SARS-CoV-2 Recombinant Spike Protein Constructs**

Linear diagram of the full-length SARS-CoV-2 spike (S) proteins based on the protein sequences of the Alpha, Gamma, Delta, and Delta Plus variants. Structural elements include the cleavable signal sequence (SS, white), N-terminal domain (NTD, blue), receptor binding domain (RBD, green), subdomains 1 and 2 (SD1 and SD2, cyan), S2 cleavage site (S2′), fusion peptide (FP, red), heptad repeat 1 (HR1, yellow), central helix (CH, orange), heptad repeat 2 (HR2, purple), transmembrane domain (TM, black), and cytoplasmic tail (CT, white). Amino acid changes from the prototype rS protein sequence (rS-WU1) are shown in black text underneath the linear diagram. The native furin cleavage site was mutated (RRAR to QQAQ) to resist proteolytic cleavage and

two proline mutations were also introduced to increase stability; these mutations are noted in red text above the linear diagram.

Figure S4: SARS-CoV-2 neutralization antibody titers

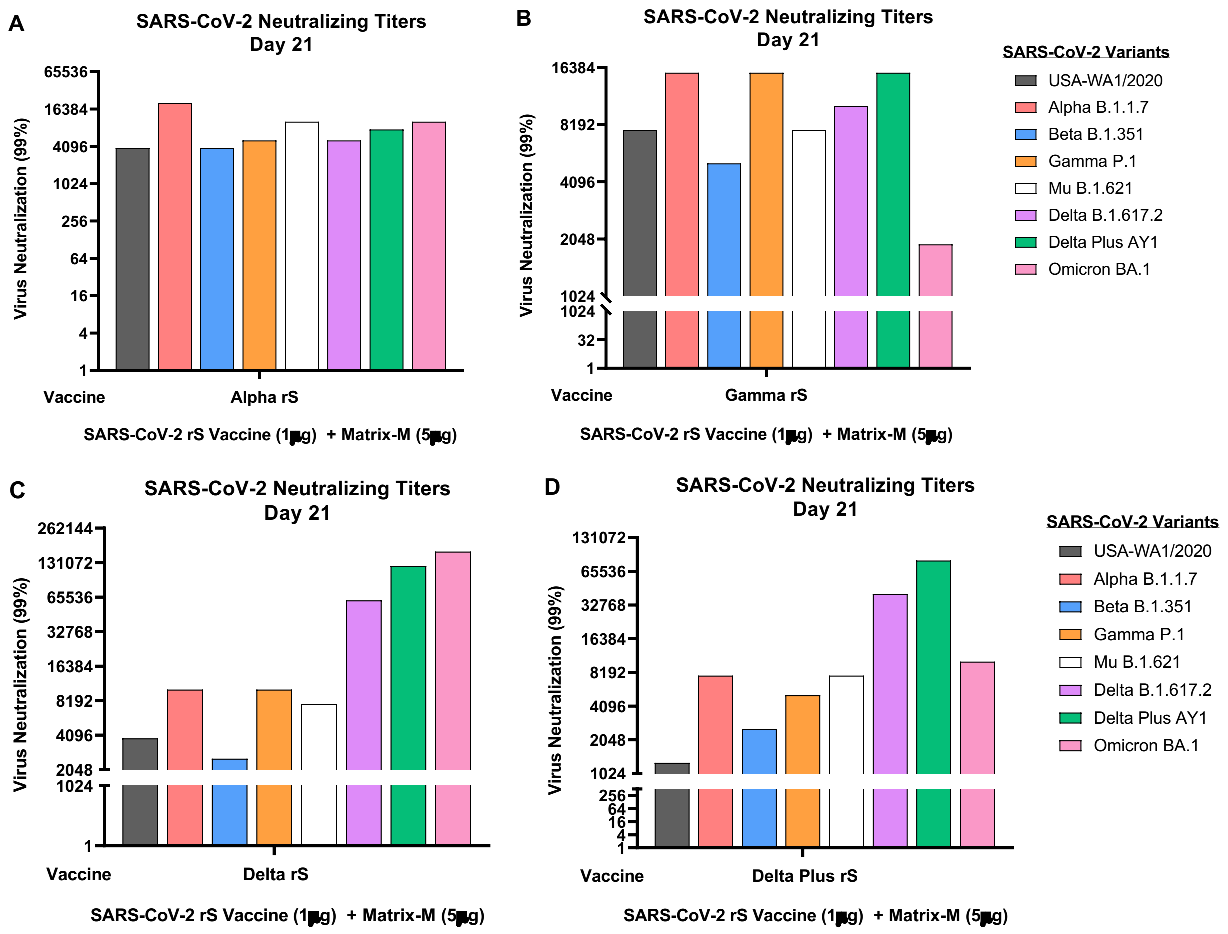


**Figure S4: SARS-CoV-2 neutralization antibody titers**

SARS-CoV-2 neutralization antibody titers in sera collected on Day 21 from animals (n= 20/group) immunized with SARS-CoV-2 (**A**) Alpha rS, (**B**) Gamma rS, (**C**) Delta rS, or (**D**) Delta Plus rS were determined using a CPE assay. Sera were pooled and evaluated for their ability to neutralize SARS-CoV-2 USA-WA1, Alpha, Beta, Gamma, Mu, Delta, Delta Plus, or Omicron BA1 variants (n=2 per sample). Bars indicate pooled serum titer from each group.

Figure S5: SARS-CoV-2 hACE2 receptor binding inhibition titers

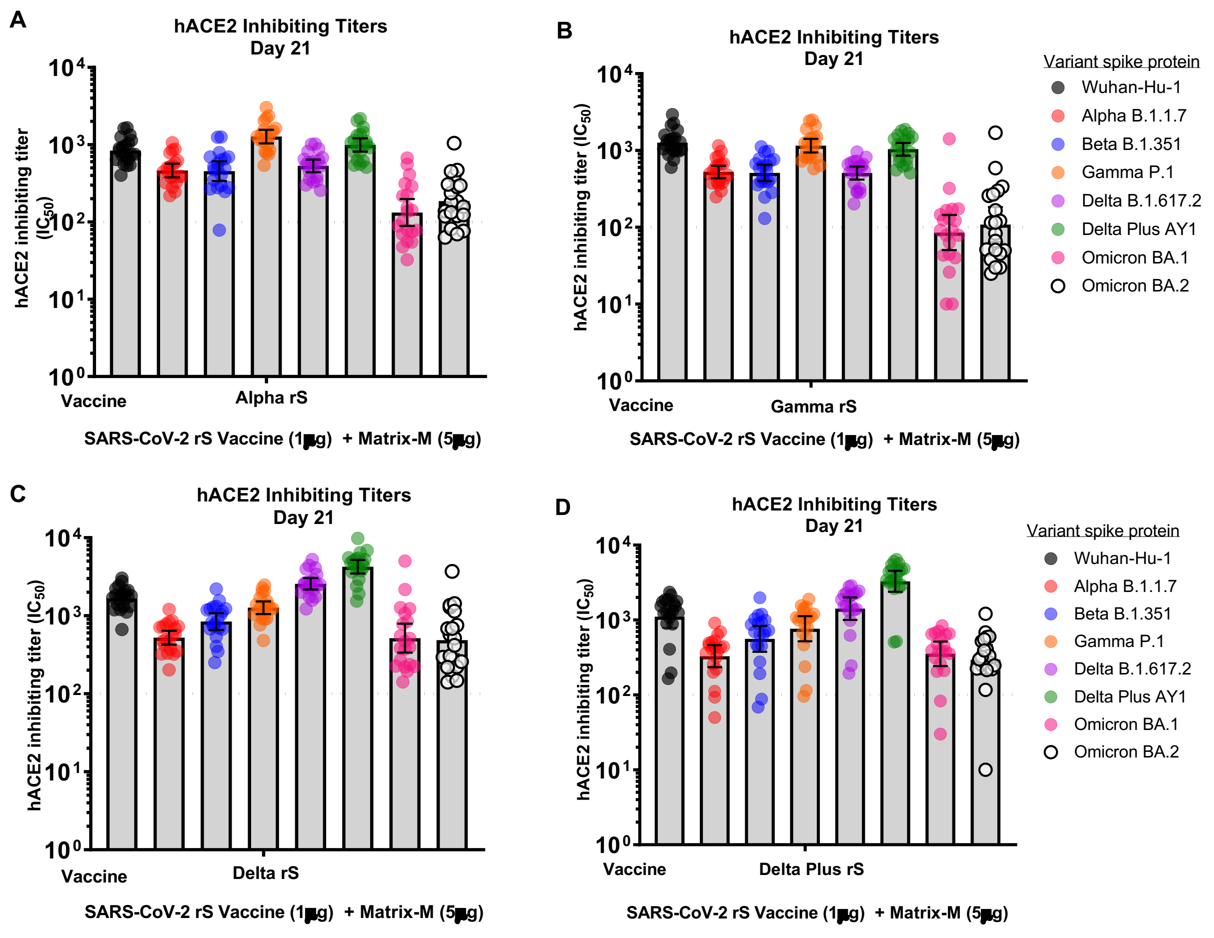


**Figure S5: SARS-CoV-2 hACE2 receptor binding inhibition titers**

Functional antibody titers capable of disrupting binding between the SARS-CoV-2 receptor hACE2 and Wuhan-Hu-1, Alpha, Beta, Gamma, Delta, Delta Plus, Omicron BA1 or Omicron BA2 spike protein were measured in sera collected on Day 21 from animals immunized with SARS-CoV-2 (**A**) Alpha rS, (**B**) Gamma rS, (**C**) Delta rS, or (**D**) Delta Plus rS. Data points indicate geometric mean titer from each group (n=20/group). Bars indicate the geometric mean titer (GMT) and error bars represent 95% confidence interval (CI) for each group. Individual animal titers are indicated with colored symbols.
