# Supplementary material for: Immunogenicity and protection of a variant nanoparticle vaccine that confers broad neutralization against SARS-CoV-2 variants
Source: Nat Commun. 2023 Feb 28;14:1130. doi: 10.1038/s41467-022-35606-6 (PMC9972327; doi:10.1038/s41467-022-35606-6)
Supplement: Supplementary file 2 — Reporting Summary [file 41467_2022_35606_MOESM2_ESM.pdf]

nature portfolio

Corresponding author(s): Matthew B. Frieman

Last updated by author(s): Nov 29, 2022

Reporting Summary

Nature Portfolio wishes to improve the reproducibility of the work that we publish. This form provides structure for consistency and transparency in reporting. For further information on Nature Portfolio policies, see our Editorial Policies and the Editorial Policy Checklist.

Statistics

For all statistical analyses, confirm that the following items are present in the figure legend, table legend, main text, or Methods section.

n/a	Confirmed	
☐	☒	The exact sample size (n) for each experimental group/condition, given as a discrete number and unit of measurement
☐	☒	A statement on whether measurements were taken from distinct samples or whether the same sample was measured repeatedly
☐	☒	The statistical test(s) used AND whether they are one- or two-sided<br>*Only common tests should be described solely by name; describe more complex techniques in the Methods section.*
☐	☒	A description of all covariates tested
☐	☒	A description of any assumptions or corrections, such as tests of normality and adjustment for multiple comparisons
☐	☒	A full description of the statistical parameters including central tendency (e.g. means) or other basic estimates (e.g. regression coefficient) AND variation (e.g. standard deviation) or associated estimates of uncertainty (e.g. confidence intervals)
☐	☒	For null hypothesis testing, the test statistic (e.g. F, t, r) with confidence intervals, effect sizes, degrees of freedom and P value noted<br>*Give P values as exact values whenever suitable.*
☒	☐	For Bayesian analysis, information on the choice of priors and Markov chain Monte Carlo settings
☒	☐	For hierarchical and complex designs, identification of the appropriate level for tests and full reporting of outcomes
☐	☒	Estimates of effect sizes (e.g. Cohen's d, Pearson's r), indicating how they were calculated

Our web collection on statistics for biologists contains articles on many of the points above.

Software and code

Policy information about availability of computer code

Data collection	*Provide a description of all commercial, open source and custom code used to collect the data in this study, specifying the version used OR state that no software was used.*
Data analysis	*Provide a description of all commercial, open source and custom code used to analyse the data in this study, specifying the version used OR state that no software was used.*

For manuscripts utilizing custom algorithms or software that are central to the research but not yet described in published literature, software must be made available to editors and reviewers. We strongly encourage code deposition in a community repository (e.g. GitHub). See the Nature Portfolio guidelines for submitting code & software for further information.

Data

Policy information about availability of data

All manuscripts must include a data availability statement. This statement should provide the following information, where applicable:

- Accession codes, unique identifiers, or web links for publicly available datasets
- A description of any restrictions on data availability
- For clinical datasets or third party data, please ensure that the statement adheres to our policy

All data is available and datasets uploaded and available upon publication.

Human research participants

Policy information about studies involving human research participants and Sex and Gender in Research.

Reporting on sex and gender	*Use the terms sex (biological attribute) and gender (shaped by social and cultural circumstances) carefully in order to avoid confusing both terms. Indicate if findings apply to only one sex or gender; describe whether sex and gender were considered in study design whether sex and/or gender was determined based on self-reporting or assigned and methods used. Provide in the source data disaggregated sex and gender data where this information has been collected, and consent has been obtained for sharing of individual-level data; provide overall numbers in this Reporting Summary. Please state if this information has not been collected. Report sex- and gender-based analyses where performed, justify reasons for lack of sex- and gender-based analysis.*
Population characteristics	*Describe the covariate-relevant population characteristics of the human research participants (e.g. age, genotypic information, past and current diagnosis and treatment categories). If you filled out the behavioural & social sciences study design questions and have nothing to add here, write "See above."*
Recruitment	*Describe how participants were recruited. Outline any potential self-selection bias or other biases that may be present and how these are likely to impact results.*
Ethics oversight	*Identify the organization(s) that approved the study protocol.*

Note that full information on the approval of the study protocol must also be provided in the manuscript.

Field-specific reporting

Please select the one below that is the best fit for your research. If you are not sure, read the appropriate sections before making your selection.

☒ Life sciences ☐ Behavioural & social sciences ☐ Ecological, evolutionary & environmental sciences

For a reference copy of the document with all sections, see nature.com/documents/nr-reporting-summary-flat.pdf

Life sciences study design

All studies must disclose on these points even when the disclosure is negative.

Sample size	All sample size is stated in the text of the manuscript
Data exclusions	N/A
Replication	All replicate numbers are stated in the text of the manuscript
Randomization	N/A
Blinding	N/A

Reporting for specific materials, systems and methods

We require information from authors about some types of materials, experimental systems and methods used in many studies. Here, indicate whether each material, system or method listed is relevant to your study. If you are not sure if a list item applies to your research, read the appropriate section before selecting a response.

Materials & experimental systems

n/a	Involved in the study	
☐	☒	Antibodies
☐	☒	Eukaryotic cell lines
☒	☐	Palaeontology and archaeology
☐	☒	Animals and other organisms
☒	☐	Clinical data
☒	☐	Dual use research of concern

Methods

n/a	Involved in the study	
☒	☐	ChIP-seq
☐	☒	Flow cytometry
☒	☐	MRI-based neuroimaging

Antibodies

Antibodies used	Surface Staining (T folicular helper cell FLOW, all from BD Biosciences, San Jose, CA)<br>BV650-conjugated anti-CD3 - Cat#: 564378<br>APC-H7-conjugated anti-CD4 - Cat#: 552051<br>FITC-conjugated anti-CD8 - Cat#: 553030<br>Percp-cy5.5-conjugated anti-CXCR5 - Cat#: 560528<br>APC-conjugated anti-PD-1 - Cat#: 562671<br>Alexa Fluor 700-conjugated anti-CD19 - Cat#: 557958<br>PE-conjugated anti-CD49b - Cat#: 558759<br><br>Surface Staining (Germinal Center Flow, all from BD Biosciences, San Jose, CA)<br>FITC-conjugated anti-CD3 - Cat#: 553062<br>PerCP-Cy5.5-conjugated anti-B220 - Cat#: 553093<br>APC-conjugated anti-CD19 - Cat#: 550992<br>PE-cy7-conjugated anti-CD95 - Cat#: 557653<br>BV421-conjugated anti-GL7 - Cat#: 562967<br><br>Intracellular Staining (Mouse Flow Samples, all from BD Biosciences, San Jose, CA)<br>Murine antibodies against CD3 (BV650) - Cat#: 564378<br>CD4 (APC-H7) - Cat#: 552051<br>CD8 (FITC) - Cat#: 553030<br>CD44 (Alexa Fluor 700) - Cat#: 560567<br>CD62L (PE) (BD Pharmingen, CA) - Cat#: 553151<br>PerCP-Cy5.5-conjugated anti-IFN-γ - Cat#: 560660<br>BV421-conjugated anti-IL-2 - Cat#: 562969<br>PE-Cy7-conjugated anti-TNF-α - Cat#: 557644<br>APC-conjugated anti-IL-4 - Cat#: 554436<br><br>Intracellular Staining (Baboon Flow Samples, all from BD Biosciences)<br>BV650-conjugated anti-CD3 - Cat#: 563916<br>APC-H7-conjugated anti-CD4 - Cat#: 560837<br>FITC-conjugated anti-CD8 - Cat#: 561948<br>BV421-conjugated anti-IL-2 - Cat#: 564164<br>PerCP-Cy5.5-conjugated anti-IFN-γ - Cat#: 560742<br>PE-Cy7-conjugated anti-TNF-α - Cat#: 557647<br>APC-conjugated anti-IL-5 - Cat#: 554396<br>BV711-conjugated anti-IL-13 - Cat#: 564288
Validation	N/A

Eukaryotic cell lines

Policy information about cell lines and Sex and Gender in Research

Cell line source(s)	All cell lines sourced from ATCC and described in methods
Authentication	All cell lines sourced from ATCC and validated by ATCC
Mycoplasma contamination	Tested for all cell lines
Commonly misidentified lines (See ICLAC register)	N/A

Animals and other research organisms

Policy information about studies involving animals; ARRIVE guidelines recommended for reporting animal research, and Sex and Gender in Research

Laboratory animals	BalbC mice used in the study sourced from Charles River at 8-10 weeks old.<br>Olive Baboons used in this study were housed at Oklahoma Health Science Center and were 10-16 years old at study initiation.
Wild animals	N/A
Reporting on sex	Balb/c mice: All mice used were female<br>Olive baboons: 4 female, 5 male; each experimental group included at least one male and one female
Field-collected samples	N/A

Ethics oversight | N/A

Note that full information on the approval of the study protocol must also be provided in the manuscript.

Flow Cytometry

Plots

Confirm that:

- [x] The axis labels state the marker and fluorochrome used (e.g. CD4-FITC).
- [x] The axis scales are clearly visible. Include numbers along axes only for bottom left plot of group (a 'group' is an analysis of identical markers).
- [x] All plots are contour plots with outliers or pseudocolor plots.
- [x] A numerical value for number of cells or percentage (with statistics) is provided.

Methodology

Sample preparation

For surface staining, murine splenocytes were first incubated with an anti-CD16/32 antibody to block the Fc receptor. To characterize T follicular helper cells (Tfh), splenocytes were incubated with the following antibodies or dye: BV650-conjugated anti-CD3, APC-H7-conjugated anti-CD4, FITC-conjugated anti-CD8, Percp-cy5.5-conjugated anti-CXCR5, APC-conjugated anti-PD-1, Alexa Fluor 700-conjugated anti-CD19, PE-conjugated anti-CD49b (BD Biosciences, San Jose, CA) and the yellow LIVE/DEAD® dye (Life Technologies, NY). To stain germinal center (GC) B cells, splenocytes were labeled with FITC-conjugated anti-CD3, PerCP-Cy5.5-conjugated anti-B220, APC-conjugated anti-CD19, PE-cy7-conjugated anti-CD95, and BV421-conjugated anti-GL7 (BD Biosciences) and the yellow LIVE/DEAD® dye (Life Technologies, NY).

For intracellular cytokine staining (ICCS) of murine splenocytes, cells were cultured in a 96-well U-bottom plate at 2 × 106 cells per well. The cells were stimulated with rS-WU1 or rS-Beta spike protein. The plate was incubated 6 h at 37°C in the presence of BD GolgiPlug™ and BD GolgiStop™ (BD Biosciences) for the last 4 h of incubation. Cells were labeled with murine antibodies against CD3 (BV650), CD4 (APC-H7), CD8 (FITC), CD44 (Alexa Fluor 700), and CD62L (PE) (BD Pharmingen, CA) and the yellow LIVE/DEAD® dye. After fixation with Cytofix/Cytoperm (BD Biosciences), cells were incubated with PerCP-Cy5.5-conjugated anti-IFN-γ, BV421-conjugated anti-IL-2, PE-Cy7-conjugated anti-TNF-α, and APC-conjugated anti-IL-4 (BD Biosciences).

For ICS of baboon PBMCs, PBMCs collected at the timepoints listed in Figure 5A were stimulated as described above with rS-WU1 or rS-Beta. Cells were labelled with human/NHP antibodies BV650-conjugated anti-CD3, APC-H7-conjugated anti-CD4, FITC-conjugated anti-CD8, BV421-conjugated anti-IL-2, PerCP-Cy5.5-conjugated anti-IFN-γ, PE-Cy7-conjugated anti-TNF-α, APC-conjugated anti-IL-5, BV711-conjugated anti-IL-13 (BD Biosciences), and the yellow LIVE/DEAD® dye.

Instrument

All stained samples were acquired using a LSR-Fortessa or a FACSymphony flow cytometer (Becton Dickinson, San Jose, CA)

Software

The data were analyzed with FlowJo software version Xv10 (Tree Star Inc., Ashland, OR)

Cell population abundance

Cell population abundance shown in each figure.

Gating strategy

Mouse study
T Follicular Helper Cells: Gated on live “CD3+CD4+CD19-“ CD4+ T cells.
Memory B cells: Gated on live “CD19+B220+CD3-“ B cells.

Intracellular Cytokine Staining: Gated on live “CD3+CD4+CD44hiCD62Llow” effector CD4+ T cells. Or “CD3+CD8+ CD44hiCD62Llow” effector CD8+ T cells.

Baboon Study:
Intracellular Cytokine Staining: Gated on live “CD3+CD4+” CD4+ T cells

- [ ] Tick this box to confirm that a figure exemplifying the gating strategy is provided in the Supplementary Information.
